# A primary hepatopancreatic cell platform enables cellular dissection of glucose metabolism and endocrine-metabolic regulation in *Litopenaeus vannamei*

**DOI:** 10.3389/fendo.2026.1901016

**Published:** 2026-08-21

**Authors:** Xiaowei Song, Zijie Liu, Jiawei Liu, Su Liu, Lin Song, Hu Duan, Yi Gao

**Affiliations:** 1 School of Marine Science and Engineering, Qingdao Agricultural University, Qingdao, China; 2 College of Biological Engineering, Qingdao University of Science and Technology, Qingdao, China; 3 Wuqiong Food Co., Ltd., Raoping, China; 4 College of Marine and Environmental Sciences, Tianjin University of Science and Technology, Tianjin, China

**Keywords:** endocrine-metabolic regulation, glucose metabolism, insulin-like signaling, *Litopenaeus vannamei*, primary hepatopancreatic cells

## Abstract

The lack of robust continuous cell lines remains a major bottleneck limiting cell-level mechanistic dissection of endocrine and metabolic regulation in crustaceans. In this context, experimentally tractable primary cell systems provide a practical and physiologically relevant strategy for interrogating regulatory processes under controlled conditions. Here, we established and functionally validated a primary hepatopancreatic cell platform from the Pacific white shrimp *Litopenaeus vannamei* for investigating glucose metabolism and endocrine-metabolic regulation. Morphological characterization and systematic optimization of key culture parameters showed that the isolated cell population retained representative features of major hepatopancreatic cell types and could be maintained under short-term culture conditions suitable for functional analyses. Using this system, we showed that primary hepatopancreatic cells rapidly sensed exogenous glucose and initiated a coordinated metabolic response characterized by enhanced glucose transport and glycolysis-related transcription, together with suppression of gluconeogenesis-related genes. Exogenous insulin further amplified intracellular glucose accumulation and reinforced this glucose-utilizing transcriptional program, indicating that the platform preserves hormone-responsive metabolic properties *in vitro*. Conversely, starvation-mimicking conditions induced a distinct adaptive response, marked by reduced intracellular glucose availability, repression of glycolysis-related genes, and activation of gluconeogenesis-related transcription. Linsitinib treatment substantially blunted glucose responsiveness and shifted the transcriptional profile toward impaired glucose utilization. To extend the applicability of the platform beyond acute *in vitro* stimulation, *in vivo* endocrine perturbations were coupled with subsequent *in vitro* glucose-loading assays. Primary cells derived from insulin-degrading enzyme (IDE)-suppressed shrimp displayed enhanced glucose accumulation, whereas prolonged insulin exposure produced a delayed glucose-response profile, indicating an altered temporal response to glucose loading. In addition, optimized cytochemical staining enabled visualization of glycogen and lipid storage differences under distinct nutritional states, providing complementary phenotypic readouts of cellular energy status. Collectively, this study establishes a controllable and expandable primary hepatopancreatic cell platform for dissecting nutrient sensing, glucose metabolic homeostasis, and endocrine-metabolic regulation in *L. vannamei*. This system provides a valuable cellular tool for crustacean endocrinology and metabolism research and offers a useful framework for future functional studies in economically important crustacean species.

## 1 Introduction

Crustaceans, particularly shrimp and crabs, represent one of the most economically important groups in global aquaculture (1). In farmed crustaceans, rapid growth and efficient nutrient utilization are major production targets, both of which depend closely on coordinated metabolic regulation. Metabolic homeostasis therefore provides a fundamental physiological basis for nutrient use, energy balance, and growth performance in these species (2).

Although substantial progress has been made in crustacean metabolic research, the mechanisms governing metabolic homeostasis in crustaceans remain less well defined than those in vertebrates and established insect models (3). Glucose metabolism is a central component of energy metabolism and is closely linked to nutrient utilization, cellular signaling, and physiological regulation (4, 5). Previous studies have shown that glucose loading, exogenous insulin stimulation, starvation, and changes in endogenous regulators such as insulin-like peptides (ILPs) and crustacean hyperglycemic hormone (CHH) can markedly affect hemolymph glucose homeostasis, glucose clearance, and the expression of glucose metabolism-related genes in crustaceans (6–8). These findings indicate that glucose metabolism in crustaceans is not merely a nutrient-utilization process, but is closely integrated with endocrine regulation and systemic energy allocation. However, current knowledge has been derived mainly from whole-animal experiments, including *in vivo* injection, long-term feeding trials, starvation treatment, and *in vivo* gene knockdown (4, 9). Although these approaches are valuable for assessing systemic metabolic responses, they often require large numbers of animals and extended experimental periods, and are readily affected by environmental conditions and inter-individual variation.

Stable, controllable, and reproducible *in vitro* cell models are therefore important for dissecting the cellular basis of metabolic regulation in crustaceans (10). However, the development of crustacean cell culture systems, particularly in penaeid shrimp, has long been challenging (11, 12). Since the 1990s, primary cultures have been attempted using various tissue sources, including the ovary, embryos, limb buds, lymphoid organs, and hemocytes (13–15), and efforts have also been made to achieve long-term culture or cell immortalization (16). Nevertheless, no widely recognized and robust immortalized cell line is currently available for shrimp (10). In this context, primary cell culture remains one of the most practical systems for *in vitro* functional studies in shrimp (17). For metabolic and endocrine research, such systems provide a useful means to examine direct cellular responses to nutritional or hormonal stimuli under controlled conditions, while reducing the influence of whole-animal physiological status, environmental variation, and individual differences. Establishing a primary cell system suitable for glucose metabolism and insulin-like regulation would therefore facilitate more precise mechanistic analysis at the cellular level.

Among candidate tissues, the hepatopancreas is one of the major metabolic organs in crustaceans (18). As the midgut gland, it is responsible for digestive enzyme secretion, nutrient absorption, storage, transport, and metabolic regulation, and is often considered a multifunctional organ that partially integrates roles analogous to those of the vertebrate intestine, liver, and pancreas (18, 19). The decapod hepatopancreas has a well-defined cellular composition and a highly specialized functional architecture, making it an important tissue for studying nutrient metabolism, endocrine regulation, and environmental responses (18, 20). Previous studies have attempted to establish primary hepatopancreatic cell cultures in shrimp and used them to investigate white spot syndrome virus (WSSV) infection and cytopathic changes (21). Primary hepatopancreatic cells have also been applied to functional studies of endocrine and nutritional factors. For example, Wei et al. used feeding and *in vitro* primary hepatopancreatic cell assays to examine leucine-mediated regulation of TOR signaling and metabolic gene expression in *L. vannamei* (22). Luo et al. and Chen et al. further combined primary hepatopancreatic cell culture with *in vivo* injection to assess the effects of MIH- and VIH-related CHH-family peptides on hepatopancreatic vitellogenin expression (23, 24). Together, these studies support the utility of hepatopancreas-derived cells for *in vitro* functional assays. However, previous studies have generally used primary hepatopancreatic cells as auxiliary tools for pathogen-related investigations or for validating the effects of specific nutritional or endocrine factors (21, 25). Systematic optimization of culture conditions and the development of these cells as a platform for glucose metabolism and endocrine-metabolic studies remain limited.

In the present study, we used *L. vannamei*, one of the most economically valuable farmed shrimp species worldwide, to develop a functionally validated primary hepatopancreatic cell platform for investigating glucose metabolism and endocrine–metabolic regulation. Rather than simply establishing a primary hepatopancreatic cell culture system, we sought to extend its functional application by integrating culture optimization, metabolic phenotyping, assessment of endocrine and pharmacological responsiveness, and *in vivo* to *in vitro* functional validation. We first optimized key culture conditions and established a metabolic phenotyping framework combining dynamic intracellular glucose measurements with the expression of glucose metabolism-related genes. The optimized system was then evaluated under glucose loading, exogenous insulin treatment, starvation-mimicking conditions, and pharmacological treatment with linsitinib (OSI-906). In addition, by combining *in vivo* insulin-degrading enzyme (IDE) gene knockdown and prolonged exogenous insulin exposure with subsequent *in vitro* glucose-loading assays, we examined the applicability of this system for cellular functional validation under different endocrine backgrounds. Periodic acid–Schiff and Oil Red O staining were further incorporated as complementary phenotypic readouts of cellular glycogen and lipid storage. Collectively, these approaches extend the application of primary hepatopancreatic cells beyond their previous use as auxiliary experimental models and provide a tractable platform for investigating nutrient sensing, glucose metabolism, and insulin-like endocrine regulation in shrimp.

## 2 Materials and methods

### 2.1 Experimental animals

Healthy *L. vannamei* were obtained from a commercial aquaculture farm in Haiyang, Shandong Province, China. Before the experiments, shrimp were acclimated for at least 2 weeks in a recirculating seawater aquaculture system at the College of Marine Science and Engineering, Qingdao Agricultural University. Shrimp were maintained in approximately 1 m³ tanks at a stocking density of 200–300 individuals/m³. Water temperature and salinity were maintained at 23.0 ± 1.0 °C and 20.0 ± 1.0‰, respectively, with continuous aeration. Shrimp were fed a commercial pelleted diet (Tongwei, Chengdu, China) three times daily at 07:00, 15:00, and 23:00. Fresh seawater was replaced daily throughout the experiment to maintain stable water quality.

All animal husbandry and experimental procedures were approved by the Animal Care Committee of Qingdao Agricultural University (approval no. 2018-192).

### 2.2 Isolation and primary culture of hepatopancreatic cells from *L. vannamei*

The primary hepatopancreatic cell culture protocol for *L. vannamei* was adapted from methods previously established in our laboratory (26, 27), with modifications according to the aims of the present study. Glucose-free Leibovitz’s L-15 medium (PM151014, Procell, Wuhan, China) was used as the basal medium, and the detailed medium composition is provided in **Table 1**.

**Table 1:** Composition of the basal L-15 medium used for primary hepatopancreatic cell culture.

Components	Amount	Supplier
HEPES	500 μL	Solarbio
NaHCO_3_	0.06 g	Solarbio
Yeast extract	0.25 g	Procell
Penicillin	100 U/mL	Solarbio
Streptomycin	100 μg/mL	Solarbio
Leibovitz’s L-15 medium	Up to 50 mL	Procell

The pH of the medium was adjusted to 7.2 and the medium was sterilized through a 0.22 μm filter before use. The culture temperature was maintained at 26 °C.

All cell isolation procedures were performed aseptically in a biosafety cabinet. Before the experiment, the working area was sterilized by ultraviolet irradiation for 1 h. To reduce microbial contamination from the body surface and digestive tract, shrimp were maintained in sterile seawater containing tetracycline hydrochloride (50 mg/L) for 10–12 h before cell isolation. For each experiment, eight healthy shrimp were randomly selected and surface-disinfected with 75% ethanol. The shrimp were then dissected aseptically, and the hepatopancreas was excised and immediately transferred to sterile culture dishes. Hepatopancreatic tissues were minced into approximately 1 mm³ fragments using sterile scalpels, scissors, and forceps. The fragments were washed twice with antibiotic-containing L-15 medium to remove residual hemolymph and tissue debris, followed by the addition of fresh culture medium. The tissue suspension was gently pipetted to promote cell release and dispersion, and then filtered through a sterile 150-mesh cell strainer to obtain a primary hepatopancreatic cell suspension.

The suspension was centrifuged at low speed (1,000 rpm, 5 min), and the resulting cell pellet was resuspended in fresh culture medium. Cell density was determined using an automated cell counter (Countstar, Shanghai, China) and adjusted to 5-6 × 10^5^ cells/mL. The cell suspension was seeded into 24-well plates and cultured in a constant-temperature incubator at 26 °C.

### 2.3 Optimization of primary hepatopancreatic cell culture conditions

To optimize the *in vitro* culture conditions for primary hepatopancreatic cells from *L. vannamei*, the effects of medium pH, NaCl supplementation, fetal bovine serum (FBS) concentration, and shrimp muscle extract (SME) supplementation on cell viability were evaluated using the isolation and culture protocol described in Section 2.2. Cell viability was assessed using an Enhanced Cell Counting Kit-8 (CCK-8; Seven Innovation, Beijing, China).

For the pH, NaCl, and FBS optimization experiments, isolated primary hepatopancreatic cells were adjusted to 5-6 × 10^5^ cells/mL and seeded into 96-well plates at 100 μL/well. Cells were cultured at 26 °C for 12 h, after which 10 μL of CCK-8 reagent was added to each well. After an additional 1 h of incubation, absorbance at 450 nm was measured using a microplate reader. Cell-free blank wells containing the corresponding culture medium and the same amount of CCK-8 reagent as the experimental wells, but without cells, were processed in parallel under the same conditions and used for background absorbance correction. All optimization experiments were performed with four biological replicates, each containing four technical replicates.

#### 2.3.1 Effect of medium pH on cell viability

To determine the effect of medium pH on cell viability, the pH of basal L-15 medium was adjusted to 7.1, 7.2, 7.3, 7.4, and 7.5 using HCl or NaOH. The prepared media were sterilized by filtration through a 0.22 μm membrane before use. Primary hepatopancreatic cells were cultured in media with different pH values for 12 h, and cell viability was then determined using the CCK-8 assay.

#### 2.3.2 Effect of NaCl supplementation on cell viability

To evaluate the effect of NaCl supplementation, basal L-15 medium was supplemented with NaCl at final additional levels of 0‰, 2‰, 4‰, 6‰, and 8‰, with all other medium components kept constant. The media were sterilized by filtration through a 0.22 μm membrane before use. Primary hepatopancreatic cells were cultured in media with different NaCl supplementation levels for 12 h, followed by CCK-8-based viability assessment.

#### 2.3.3 Effect of FBS concentration on cell viability

To optimize serum supplementation, FBS (Procell, Wuhan, China) was added to basal L-15 medium at final concentrations of 5%, 10%, 15%, 20%, and 25%. After filtration through a 0.22 μm membrane, the prepared media were used for primary hepatopancreatic cell culture. Cells were cultured under the different FBS conditions for 12 h, and cell viability was assessed using the CCK-8 assay.

#### 2.3.4 Effect of SME supplementation on cell viability

To prepare SME, healthy *L. vannamei* were surface-disinfected with 75% ethanol and dissected aseptically to collect muscle tissue. The tissue was washed three times with ice-cold 1 × PBS (Abcell, Beijing, China) to remove residual hemolymph and debris, and then homogenized thoroughly on ice. After centrifugation to remove insoluble tissue debris, the supernatant was collected and sterilized by filtration through a 0.22 μm membrane. The resulting filtrate was used as the SME stock solution.

For the SME supplementation assay, isolated primary hepatopancreatic cells were adjusted to 5-6 × 10^5^ cells/mL and seeded into 96-well plates at 90 μL/well. Subsequently, 10 μL of SME working solution was added to each well to obtain final SME concentrations of 0%, 1%, 2%, 3%, and 4% (v/v). After 12 h of culture, cell viability was determined using the CCK-8 assay.

### 2.4 *In vitro* glucose-loading experiment in primary hepatopancreatic cells

Primary hepatopancreatic cells from *L. vannamei* were isolated and cultured as described in Section 2.2. The cell suspension was seeded into 24-well plates at 380 μL/well and pre-cultured at 26 °C for 24 h to allow adaptation to the *in vitro* culture conditions. After pre-culture, 20 μL of glucose solution (50 mg/mL) was added to each well in the glucose-treated group, yielding a final glucose concentration of 2.5 mg/mL. The control group received an equal volume of glucose-free L-15 medium. The glucose-loading concentration was determined based on preliminary experiments and relevant previous studies.

The time at which glucose or control medium was added was defined as 0 min. Cell samples were collected at 0, 10, 20, 30, 40, and 50 min using independent parallel wells for each time point. During sampling, the cell suspension was centrifuged to collect the cell pellet. After removal of the supernatant, cells were washed twice with ice-cold PBS to eliminate residual medium and extracellular glucose. The processed samples were immediately frozen in liquid nitrogen and stored at −80 °C until analysis. For intracellular glucose measurement, cell samples were resuspended in an appropriate volume of PBS, homogenized for 60 s, and centrifuged. The supernatant was collected, and intracellular glucose content was measured using a glucose assay kit (F006-1-1, Nanjing Jiancheng, Nanjing, China) according to the manufacturer’s instructions.

In parallel, cell samples collected at 20 min from the glucose-treated and control groups were used for gene expression analysis. Total RNA was extracted from primary hepatopancreatic cells using RNAiso Plus reagent (TaKaRa, Tokyo, Japan). RNA concentration and purity were determined using a NanoDrop 2000 spectrophotometer (Thermo Fisher Scientific, Waltham, MA, USA), and RNA integrity was verified by RNase-free agarose gel electrophoresis. First-strand cDNA was synthesized using the PrimeScript RT reagent Kit (TaKaRa, Tokyo, Japan) and used as the template for quantitative real-time PCR (qRT-PCR).

The expression levels of genes related to glucose transport, glycolysis, and gluconeogenesis were examined, including glucose transporter (*GLUT*), hexokinase (*HK*), phosphofructokinase (*PFK*), phosphoglycerate kinase (*PGK*), pyruvate kinase (*PK*), pyruvate carboxylase (*PC*), phosphoenolpyruvate carboxykinase (*PEPCK*), fructose-1,6-bisphosphatase (*FBP*), and glucose-6-phosphatase catalytic subunit (*G6PC*). The primer sequences used for qRT-PCR are listed in **Table 2**. qRT-PCR was performed on a QuantStudio™ 5 Real-Time PCR System (Thermo Fisher Scientific, Waltham, MA, USA) using TB Green^®^ Premix Ex Taq™ II (TaKaRa, Tokyo, Japan). The thermal cycling program consisted of initial denaturation at 95 °C for 3 min, followed by 40 cycles of 95 °C for 10 s, 55 °C for 30 s, and 72 °C for 30 s. The 18S rRNA gene was used as the internal reference, and relative gene expression was calculated using the 2^-^^ΔΔ^Ct method (28). All experiments were performed with four biological replicates, each containing four technical replicates.

**Table 2:** Primer sequences used for qRT-PCR in this study.

Primer name	Primer sequence (5′-3′)
GLUT-F	ATTGTGACCCTCTATGTGG
GLUT-R	ACTGACCCTGTACCCTTG
HK-F	GCTCGGTTTCACTTTCTC
HK-R	AGCACATCGCTTGTCCAT
PFK-F	GGCAGGCACTGTGGGTAT
PFK-R	CTCCTTCCGCTGTAATGG
PGK-F	CTTTCCTCCCTGACTGCG
PGK-R	TTGCCCTCCTCCTCTACG
PK-F	GAAGCCTCATTCAAAGCA
PK-R	CAGCATTCTGAGGCACAG
PC-F	ATTGCTCAGTCCAGCGAAG
PC-R	GAGACTGTACCAAGTCAATCCC
PEPCK-F	TGTCCAACACCATCTTCACC
PEPCK-R	TGCCCGATTCTTTGCTCC
FBP-F	AAGGAGAGGAGGTGAAGAAGC
FBP-R	CCTATGGAGACGAGGCAATC
G6PC-F	AAAGTTGGAACCTGCGGA
G6PC-R	TCTCTCCCGTCCACCAAT
IDE-F	ATGCTGCTGAGTTGGCTG
IDE-R	GTTTGTCGTTGTAACCCTTG
18S-F	TATACGCTAGTGGAGCTGGAA
18S-R	GGGGAGGTAGTGACGAAAAAT

F and R denote forward and reverse primers, respectively.

### 2.5 *In vitro* exogenous insulin treatment in primary hepatopancreatic cells

Primary hepatopancreatic cells from *L. vannamei* were isolated and cultured as described in Section 2.2. After isolation under aseptic conditions, the cell density was adjusted to 5-6 × 10^5^ cells/mL. The cell suspension was then seeded into 24-well plates at 360 μL per well and pre-cultured at 26 °C for 24 h to allow adaptation to the *in vitro* culture conditions.

Because exogenous bovine insulin has been shown to exert biological activity in the regulation of hemolymph glucose in crustaceans (7, 29), bovine insulin (G-clone, Beijing, China) was used as the exogenous insulin stimulant in this study. After pre-culture, 20 μL of glucose stock solution (50 mg/mL) was added to each well for a 10-min glucose-loading pretreatment. Subsequently, 20 μL of bovine insulin solution (1 IU/mL) was added to the insulin-treated group, yielding a final insulin concentration of 0.05 IU/mL and a final glucose concentration of 2.5 mg/mL. The control group received an equal volume of insulin vehicle. The time at which insulin or vehicle was added was defined as 0 min. Cell samples were collected at 0, 10, 20, 30, 40, and 50 min using independent parallel wells for each time point. The insulin concentration was selected based on previous reports and preliminary experiments.

During sampling, cell suspensions were centrifuged at 1,000 rpm for 5 min to collect cell pellets. After removal of the supernatant, cells were washed twice with ice-cold PBS to remove residual medium and extracellular glucose. The processed samples were resuspended in an appropriate volume of PBS, homogenized, and centrifuged. The supernatant was collected, and intracellular glucose content was determined using a glucose assay kit according to the manufacturer’s instructions.

In parallel, cell samples collected at 20 min from the insulin-treated and control groups were used for gene expression analysis. Total RNA extraction, cDNA synthesis, and qRT-PCR analysis were performed as described in Section 2.4.

### 2.6 *In vitro* starvation-mimicking treatment in primary hepatopancreatic cells

Primary hepatopancreatic cells from *L. vannamei* were isolated and cultured as described in Section 2.2. After pre-culture, cells were randomly assigned to a control group and a starvation-mimicking treatment group. To mimic sustained exogenous glucose availability, 20 μL of glucose stock solution (20 mg/mL) was added to each well in the control group every 50 min. In the starvation-mimicking treatment group, an equal volume of glucose-free L-15 medium was added at the same time points to restrict exogenous glucose supply. The treatment duration and glucose supplementation concentration were determined based on preliminary experiments.

The time at which glucose stock solution or glucose-free L-15 medium was first added was defined as 0 min. Cell samples were collected at 0, 50, 100, 150, 200, and 250 min using independent parallel wells for each time point. During sampling, cell suspensions were centrifuged at 1,000 rpm for 5 min to collect cell pellets. After removal of the supernatant, cells were washed twice with ice-cold PBS to remove residual medium and extracellular glucose. The processed samples were immediately snap-frozen in liquid nitrogen and stored at −80 °C until analysis. Intracellular glucose content was determined as described in Section 2.4.

In parallel, cell samples collected at 200 min from the control and starvation-mimicking treatment groups were used for gene expression analysis. Total RNA extraction, cDNA synthesis, and qRT-PCR analysis were performed as described in Section 2.4.

### 2.7 *In vitro* linsitinib treatment in primary hepatopancreatic cells

Primary hepatopancreatic cells from *L. vannamei* were isolated and cultured as described in Section 2.2. After pre-culture, cells were randomly assigned to a linsitinib-treated group and a vehicle control group. Linsitinib (HY-10191, MedChemExpress, NJ, USA), a small-molecule inhibitor commonly used to target insulin-like growth factor 1 receptor (IGF-1R)/insulin receptor (IR) signaling, was dissolved in dimethyl sulfoxide (DMSO; Solarbio, Beijing, China) to prepare a 10 mM stock solution and diluted with culture medium to the working concentration immediately before use. Cells in the linsitinib-treated group were pretreated with 75 nM linsitinib for 6 h, whereas those in the vehicle control group received an equivalent volume of DMSO, resulting in a final DMSO concentration of 0.00075% (v/v) in both groups. The concentration and duration of linsitinib treatment were selected based on previous reports and preliminary experiments (30, 31).

After pretreatment, both groups were subjected to glucose loading by adding 20 μL of glucose stock solution (50 mg/mL). The time at which glucose was added was defined as 0 min. Cell samples were collected at 0, 10, 20, 30, 40, and 50 min using independent parallel wells for each time point. During sampling, cell suspensions were centrifuged at 1,000 rpm for 5 min to collect cell pellets. After removal of the supernatant, cells were washed twice with ice-cold PBS to remove residual medium and extracellular glucose. Intracellular glucose content was determined as described in Section 2.4.

In parallel, cell samples collected at 20 min after glucose loading from the linsitinib-treated and vehicle control groups were used for gene expression analysis. Total RNA extraction, cDNA synthesis, and qRT-PCR analysis were performed as described in Section 2.4.

### 2.8 *In vivo* insulin-degrading enzyme knockdown combined with *in vitro* glucose-loading assay

Based on *L. vannamei* transcriptome data previously generated in our laboratory, the open reading frame (ORF) of the *IDE* gene was identified and translated into its deduced amino acid sequence. Conserved domains of IDE protein were predicted using the SMART database, and the double-stranded RNA (dsRNA) target region was designed according to the predicted domain architecture of the IDE protein.

dsRNA was synthesized by T7 *in vitro* transcription as previously described (32), with minor modifications. Briefly, the T7 promoter sequence was added to the 5′ ends of both forward and reverse primers used to amplify the interference fragment. The forward primer was 5′-TAATACGACTCACTATAGGGACTGGAGGGCTGCTGATA -3′, and the reverse primer was 5′-TAATACGACTCACTATAGGGAGGGAGGGACGGTCTAAC-3′. PCR amplification was performed using the TA-cloning plasmid containing the target fragment as the template. The 20 μL PCR mixture contained 2 μL of template, 1 μL each of forward and reverse primers, 10 μL of 2 × Phanta Max Master Mix, and nuclease-free water to a final volume of 20 μL. The PCR product was separated by 1.5% agarose gel electrophoresis, recovered, and purified. *In vitro* transcription was then performed using the TranscriptAid T7 High Yield Transcription Kit (Thermo Fisher Scientific, Waltham, USA) to synthesize *IDE* dsRNA (dsIDE). After purification, residual single-stranded RNA was removed using RNase A. Based on the interference efficiency determined in preliminary experiments, dsIDE was used at a working concentration of 1 μg/μL.

A total of 180 healthy *L. vannamei* with an average body weight of 0.94 ± 0.12 g were used for the *in vivo* RNA interference (RNAi) experiment. Shrimp were randomly assigned to a PBS control group and a dsIDE knockdown group, with three biological replicates per group and 30 shrimp per replicate. Shrimp in the PBS control group were injected with 10 μL of PBS per individual, whereas those in the dsIDE knockdown group were injected with 10 μL of dsIDE solution (1 μg/μL) per individual. All injections were administered into the abdominal muscle between the III and IV abdominal segments. To maintain sustained knockdown, injections were performed once every 96 h, for a total of five injections. The injection interval and total number of injections were selected based on preliminary trials and previous reports to maintain RNAi efficiency while minimizing stress associated with repeated injections (33–35).

At 24 h after the final injection, hepatopancreatic tissues were collected. A subset of samples was used to quantify *IDE* expression by qRT-PCR and confirm the maintenance of knockdown efficiency. The primers used for *IDE* quantification are listed in **Table 2**. Primary hepatopancreatic cells were then isolated from the PBS control and dsIDE knockdown groups as described in Section 2.2. To minimize changes associated with prolonged *in vivo* handling, cell isolation and the subsequent *in vitro* glucose-loading assay were initiated immediately after tissue collection. Cell density was adjusted to 5-6 × 10^5^ cells/mL, and the cell suspension was seeded into 24-well plates at 380 μL/well. Glucose loading was performed by adding 20 μL of glucose stock solution (50 mg/mL) to cells from both groups, yielding a final glucose concentration of 2.5 mg/mL. The time at which glucose was added was defined as 0 min. Cell samples were collected at 0, 10, 20, 30, 40, and 50 min to monitor dynamic changes in intracellular glucose content. Sampling, washing, cell lysis, and intracellular glucose measurement were performed as described in Section 2.4. The PBS control and dsIDE knockdown groups underwent identical procedures for tissue collection, cell isolation, cell density adjustment, and glucose loading, with all assays performed within the same standardized time window.

### 2.9 Glucose-response assay in primary hepatopancreatic cells after long-term exogenous insulin exposure

To evaluate the effect of long-term exogenous insulin exposure on the glucose responsiveness of primary hepatopancreatic cells from *L. vannamei*, an experimental strategy combining *in vivo* insulin treatment with an *in vitro* glucose-loading assay was established. A total of 180 healthy shrimp of similar size, with an average body weight of 0.99 ± 0.16 g, were randomly assigned to a PBS control group and a long-term insulin-treated group, with three biological replicates per group and 30 shrimp per replicate. Shrimp in the control group were injected with 10 μL of PBS per individual, whereas those in the insulin-treated group received exogenous insulin at 8.0 IU/kg body weight. Both groups were injected once every 72 h for a total of six injections over a 15-day treatment period. The injection interval and treatment duration were selected based on preliminary trials to maintain a sustained insulin exposure while minimizing stress associated with repeated injections.

To minimize potential carry-over effects from the final insulin injection on the subsequent *in vitro* assay, hepatopancreatic tissues were collected 24 h after the final treatment. Primary hepatopancreatic cells were isolated from the PBS control and long-term insulin-treated groups as described in Section 2.2. The isolated cells were adjusted to 5-6 × 10^5^ cells/mL and seeded into 24-well plates at 380 μL/well. Glucose loading was performed by adding 20 μL of glucose stock solution (50 mg/mL) to cells from both groups, yielding a final glucose concentration of 2.5 mg/mL. The time at which glucose was added was defined as 0 min, and cell samples were collected at 0, 10, 20, 30, 40, and 50 min to monitor dynamic changes in intracellular glucose concentration. Sampling, washing, cell lysis, and intracellular glucose measurement were performed as described in Section 2.4. The PBS control and long-term insulin-treated groups underwent identical procedures for tissue collection, cell isolation, cell density adjustment, and glucose loading, with all assays performed within the same standardized time window.

### 2.10 Cytochemical staining of hepatopancreatic cells

To characterize glycogen and lipid storage in hepatopancreatic cells of *L. vannamei* under different nutritional states, periodic acid-Schiff (PAS) staining and Oil Red O (ORO) staining were performed to visualize intracellular glycogen and neutral lipid deposits, respectively. Considering that hepatopancreatic cells are mostly suspended or semi-suspended and are prone to cell loss or morphological changes during fixation, washing, and staining, the procedures for cell-smear preparation, fixation, washing, and staining were optimized based on conventional PAS and ORO staining protocols to improve cell retention and staining stability.

Thirty healthy *L. vannamei* with comparable physiological status were randomly assigned to a normally fed group and a starvation-treated group. Shrimp in the normally fed group were maintained under routine feeding conditions, whereas those in the starvation-treated group were fasted for 24 h. After treatment, hepatopancreatic tissues were collected from both groups and used for cell-smear preparation.

#### 2.10.1 Preparation of hepatopancreatic cell smears

Hepatopancreatic cell suspensions were prepared from the normally fed and starvation-treated groups using the tissue dissociation and filtration procedure described in Section 2.2, with minor modifications. The collected cells were washed twice with ice-cold PBS and resuspended in a small volume of PBS. For smear preparation, 20 μL of cell suspension was placed onto the center of a clean glass slide, gently spread into a thin and uniform layer, and air-dried at room temperature before cytochemical staining.

#### 2.10.2 PAS staining

PAS staining was performed using a PAS staining kit (Solarbio, Beijing, China) with a protocol adapted for primary hepatopancreatic cell smears. To improve cell retention and preserve cellular morphology, fixation, washing, and staining durations were adjusted according to the non-adherent characteristics of the primary cells. Cell smears were fixed with 4% paraformaldehyde for 15 min and rinsed three times with distilled water. The fixed smears were incubated with PAS oxidizing solution for 15 min at room temperature, followed by two washes with distilled water for 2 min each. Schiff reagent was then added, and the smears were stained in the dark at room temperature for 20–30 min. After staining, the smears were rinsed twice with sodium sulfite solution for 2 min each and then washed with distilled water for 5 min. The smears were counterstained with hematoxylin for 1 min, rinsed with tap water, and air-dried at room temperature. Stained smears were observed and photographed under a light microscope (Axio Scope A1, Carl Zeiss AG, Germany). Quantitative analysis of staining intensity was performed using ImageJ software and expressed as average optical density (AOD), calculated by dividing the integrated optical density (IOD) by the area of positive staining.

#### 2.10.3 Oil Red O staining

ORO staining was performed using an ORO staining kit (Solarbio, Beijing, China) following an optimized protocol adapted for primary hepatopancreatic cell smears. Cell smears were fixed with 4% paraformaldehyde (Beyotime Biotech, Shanghai, China) for 15 min, rinsed three times with distilled water, and treated with 60% isopropanol for 30 s. The smears were then incubated with ORO fixative for 15 min. The ORO working solution was prepared by mixing solution A and solution B provided in the kit. After staining with the ORO working solution for 20–30 min, the smears were rinsed twice with 60% isopropanol for 30 s each, counterstained with hematoxylin for 3 min, and rinsed with tap water. After air-drying at room temperature, the smears were observed and photographed under the same light microscope described above. Quantitative analysis of ORO staining intensity was performed using the same ImageJ-based method described in Section 2.10.2, with staining intensity expressed as AOD.

### 2.11 Statistical analysis

Experimental data were obtained from at least three or four independent biological replicates, with the number of technical replicates set according to the design of each experiment. Statistical analyses were performed using SPSS 26.0. Data are presented as mean ± standard deviation (SD). Differences between two groups were analyzed using an independent-samples t-test. Differences among multiple groups were analyzed by one-way analysis of variance (one-way ANOVA), followed by Duncan’s multiple range test for *post hoc* multiple comparisons. Statistical significance was defined as *p < 0.05, and extremely significant differences were defined as **p < 0.01.

## 3 Results

### 3.1 Morphological characterization and preliminary classification of primary hepatopancreatic cells from *L. vannamei*

Based on the classical morphological framework for decapod hepatopancreatic cells, primary hepatopancreatic cells isolated from *L. vannamei* were examined by light microscopy. Multiple cell types with distinct morphological features were observed in the isolated cell suspension. Based solely on classical light microscopic criteria, some cells were tentatively classified as putative blister-like (B-like), resorptive-like (R-like), fibrillar-like (F-like), and embryonic-like (E-like) cells.

**Figure 1:**
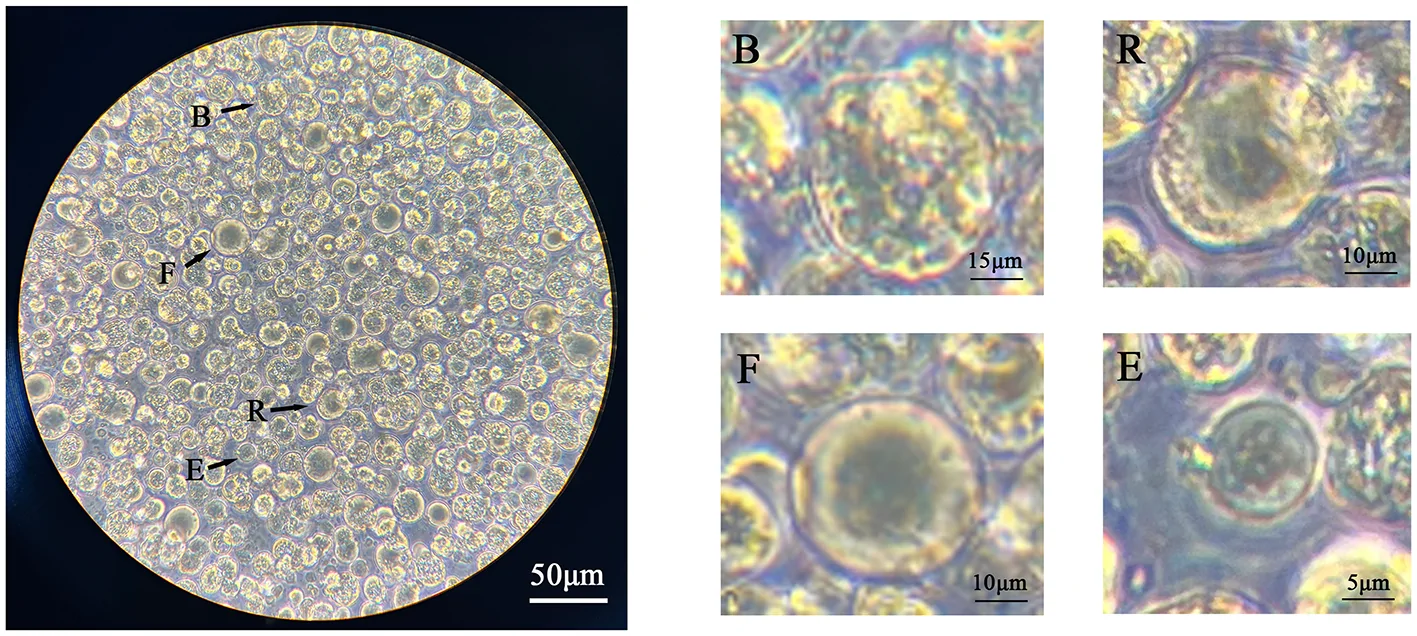
Morphological characteristics of primary hepatopancreatic cells from *L. vannamei*. The left panel shows the cell suspension observed under light microscope, displaying cell populations with distinct morphological features. The right panels show enlarged views of representative cells tentatively assigned to four morphological groups: blister-like (B-like), resorptive-like (R-like), fibrillar-like (F-like), and embryonic-like (E-like) cells. Putative B-like cells were relatively large and contained prominent vesicular or vacuole-like structures; putative R-like cells were predominantly round or nearly round with abundant cytoplasm; putative F-like cells were medium-sized with relatively dense cytoplasm; and putative E-like cells were the smallest and generally appeared round or oval. These assignments were based solely on light microscopic morphology and should be regarded as preliminary rather than definitive cell type identifications.

As shown in **Figure 1**, putative B-like cells were relatively large, with clear cell boundaries and prominent vesicular or vacuole-like structures in the cytoplasm. Putative R-like cells were predominantly round or nearly round, with well-defined outlines and abundant cytoplasm. Putative F-like cells were medium-sized and exhibited relatively dense cytoplasm, whereas putative E-like cells were the smallest and generally appeared round or oval. These observations indicate that the isolation procedure yielded a mixed primary cell population, providing a cytological basis for subsequent *in vitro* culture and functional analyses. Because the identities of these cells were not validated using specific molecular markers for individual cell types, the above designations should be regarded as preliminary classifications based on morphology rather than definitive cell type identifications. Accordingly, the metabolic and transcriptional changes observed in the subsequent experiments represent the integrated responses of the mixed primary cell population.

### 3.2 Effects of different culture conditions on the viability of primary hepatopancreatic cells from *L. vannamei*

To optimize the *in vitro* culture conditions for primary hepatopancreatic cells from *L. vannamei*, medium pH, NaCl supplementation, FBS concentration, and SME supplementation were selected as key optimization parameters based on our previous culture system (26, 27) and preliminary trials. The effects of these factors on cell viability were systematically evaluated using the CCK-8 assay.

#### 3.2.1 Effect of medium pH on cell viability

**Figure 2:**
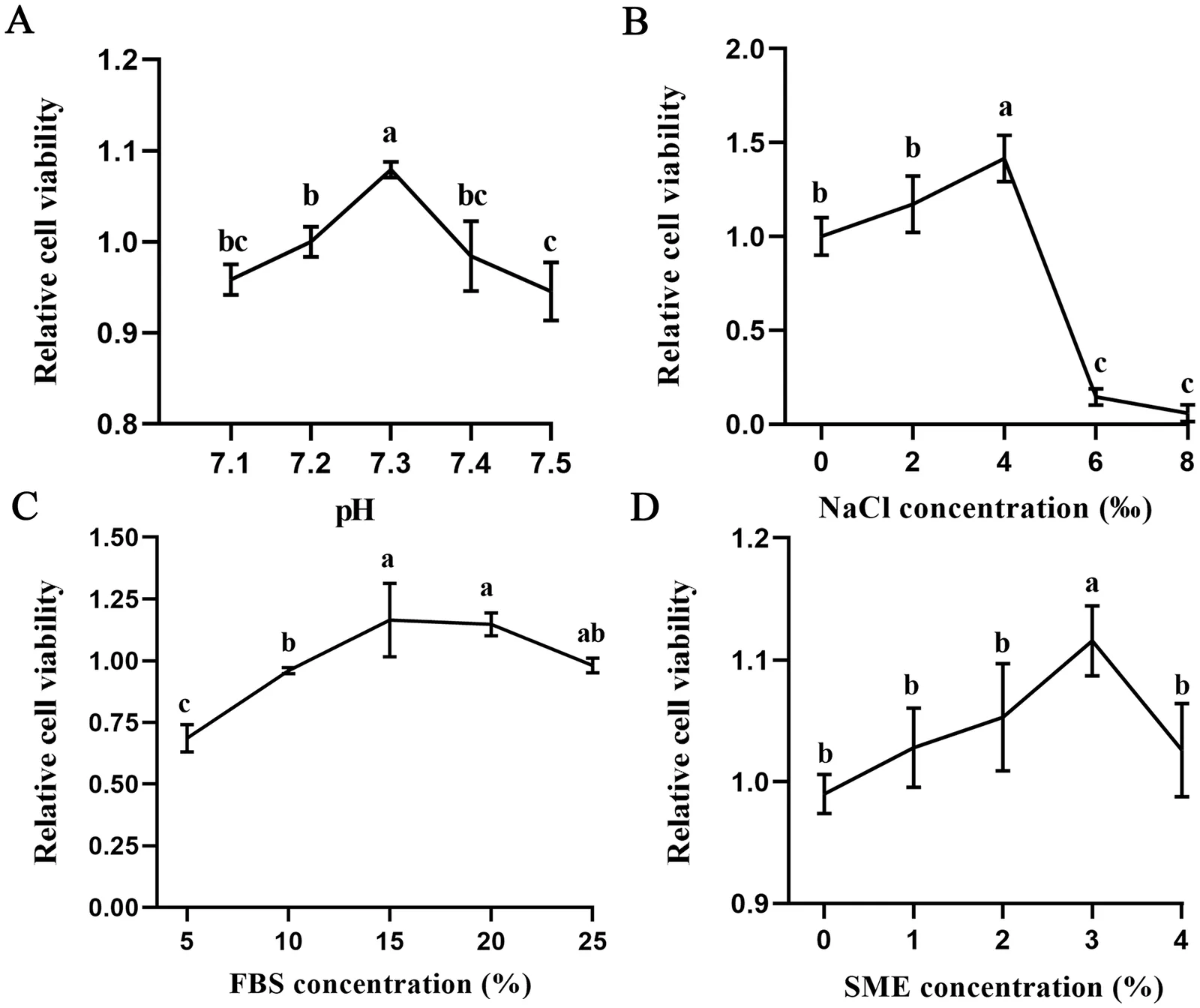
Effects of different culture conditions on the relative viability of primary hepatopancreatic cells from *L. vannamei*. **(A)** Effect of medium pH on cell viability. **(B)** Effect of NaCl supplementation level on cell viability. **(C)** Effect of FBS concentration on cell viability. **(D)** Effect of SME supplementation level on cell viability. Data are presented as the mean ± SD. Different letters indicate significant differences among groups (p < 0.05).

To determine the effect of medium pH on primary hepatopancreatic cell viability, five pH gradients were tested: 7.1, 7.2, 7.3, 7.4, and 7.5. The CCK-8 assay showed that cell viability increased as the pH rose from 7.1 to 7.3, reaching the highest level at pH 7.3, but declined when the pH was further increased to 7.4 and 7.5 (**Figure 2A**). These results indicate that primary hepatopancreatic cells from *L. vannamei* are sensitive to medium pH, with pH 7.3 being more favorable for maintaining their *in vitro* viability.

#### 3.2.2 Effect of NaCl supplementation on cell viability

Isolated cells from marine and brackish-water crustaceans are sensitive to the ionic composition and osmotic conditions of the culture medium, and NaCl supplementation is commonly used to adjust the osmotic environment of shrimp cell culture media (36). Therefore, five additional NaCl supplementation levels were tested in this study: 0‰, 2‰, 4‰, 6‰, and 8‰. Cell viability increased gradually from 0‰ to 4‰ additional NaCl and reached the highest level at 4‰, whereas further increases to 6‰ and 8‰ significantly reduced cell viability (**Figure 2B**). These results suggest that an appropriate additional NaCl supplementation supports the *in vitro* maintenance of primary hepatopancreatic cells, whereas excessive NaCl may compromise the osmotic conditions required for cell viability.

#### 3.2.3 Effect of FBS concentration on cell viability

To optimize serum supplementation, the effects of 5%, 10%, 15%, 20%, and 25% FBS on primary hepatopancreatic cell viability were compared. Cell viability increased progressively as the FBS concentration rose from 5% to 15%, reaching a relatively high level at 15% FBS (**Figure 2C**). Cell viability remained relatively high at 20% FBS, but declined when the concentration was further increased to 25%. Overall, 15%–20% FBS was more favorable for maintaining primary hepatopancreatic cells *in vitro*, and 15% FBS was selected for subsequent culture experiments.

#### 3.2.4 Effect of SME concentration on cell viability

Previous studies have shown that appropriate SME supplementation can improve the *in vitro* maintenance of shrimp primary cells and enhance cell viability (37, 38). Based on this, five final SME supplementation levels were tested: 0%, 1%, 2%, 3%, and 4%. The CCK-8 assay showed that cell viability increased gradually from 0% to 3% SME, reaching the highest level under the 3% SME condition, but declined when the SME concentration was further increased to 4% (**Figure 2D**). These results indicate that appropriate SME supplementation improves the *in vitro* viability of primary hepatopancreatic cells, whereas excessive supplementation does not further enhance cell maintenance. Therefore, 3% SME was selected as the optimal supplementation level in the present culture system.

Taken together, primary hepatopancreatic cells from *L. vannamei* showed relatively high viability under the conditions of pH 7.3, 4‰ NaCl, 15% FBS, and 3% SME. These optimized conditions provided the basis for subsequent functional experiments.

### 3.3 Establishment of a glucose challenge response model using primary hepatopancreatic cells

**Figure 3:**
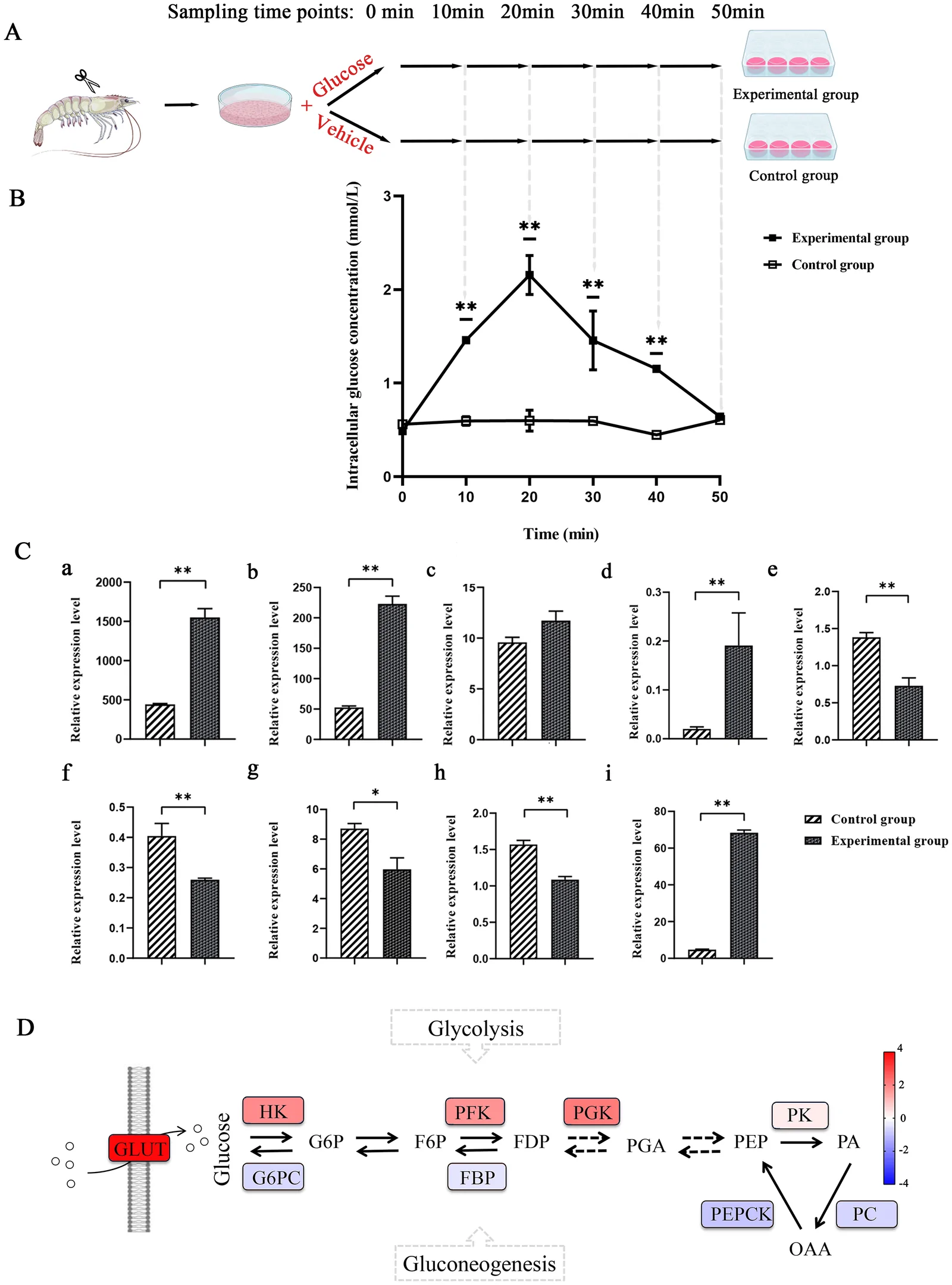
Effects of glucose challenge on glucose metabolic responses in primary hepatopancreatic cells from *L. vannamei*. **(A)** Schematic diagram of glucose treatment and sampling in primary hepatopancreatic cells. **(B)** Dynamic changes in intracellular glucose concentration from 0 to 50 min after glucose treatment. **(C)** Expression changes of glucose metabolism-related genes at 20 min after glucose treatment. **(a)** *HK*; **(b)** *PGK*; **(c)** *PK*; **(d)** *PFK*; **(e)** *PEPCK*; **(f)** *G6PC*; **(g)** *FBP*; **(h)** *PC*; **(i)** *GLUT*. **(D)** Schematic diagram of expression changes in glucose metabolism-related genes. The pathway map was adapted from the Glycolysis/Gluconeogenesis pathway (map00010) in the Kyoto Encyclopedia of Genes and Genomes (KEGG) database and integrated with the gene expression results of this study. Solid lines indicate metabolic reaction steps directly, whereas dashed lines indicate continuous reactions with intermediate steps omitted. Red indicates up-regulated gene expression, and blue indicates down-regulated gene expression; color intensity represents the log_2_ fold change relative to the control group. Data are presented as the mean ± SD. *p < 0.05, **p < 0.01.

To evaluate the applicability of the primary hepatopancreatic cell system from *L. vannamei* for *in vitro* studies of glucose metabolism, an exogenous glucose challenge was performed to establish a cellular response model. After glucose treatment, primary hepatopancreatic cells were sampled continuously at 0, 10, 20, 30, 40, and 50 min, and dynamic changes in intracellular glucose concentration were measured (**Figures 3A, B**). The results showed that intracellular glucose concentration in the glucose-treated group increased rapidly after treatment, reaching a peak of approximately 2.2 mmol/L at 20 min, and then gradually declined to a level comparable to that of the control group by 50 min. In contrast, intracellular glucose concentration in the control group remained relatively stable throughout the observation period. These results indicate that primary hepatopancreatic cells can respond rapidly to exogenous glucose stimulation and display a transient glucose accumulation profile within approximately 50 min, providing a basic model for subsequent functional experiments.

Because intracellular glucose concentration peaked at 20 min, this time point was selected for further analysis of key genes involved in glucose transport, glycolysis, and gluconeogenesis (**Figure 3C**). The examined genes included *HK*, *PGK*, *PK*, *PFK*, *PEPCK*, *G6PC*, *FBP*, *PC*, and *GLUT*. Glucose treatment markedly induced the expression of glycolysis-related genes. Specifically, *HK*, *PGK*, and *PFK* were significantly up-regulated (**Figures 3C, a, b, d**), while *PK* also showed an increasing trend (**Figures 3C, c**). In parallel, *GLUT* expression was significantly up-regulated, reaching approximately 13.5-fold that of the control group (**Figures 3C, i**). These results indicate that primary hepatopancreatic cells rapidly activated glucose transport- and glycolysis-related transcriptional responses after glucose challenge.

In contrast, the expression levels of the gluconeogenesis-related genes *PEPCK*, *G6PC*, *FBP*, and *PC* were lower than those in the control group (**Figures 3C, e–h**). Together with the schematic representation of the glucose metabolic pathway (**Figure 3D**), these results show that glucose treatment elicited a coordinated metabolic response characterized by enhanced glucose transport, up-regulation of glycolysis-related genes, and down-regulation of gluconeogenesis-related genes. Overall, this primary hepatopancreatic cell system was able to sense glucose challenge rapidly and generate a clear glucose metabolism-related transcriptional response, supporting its use for cellular-level validation of glucose metabolic regulation in *L. vannamei*.

### 3.4 Application of the primary hepatopancreatic cell system for evaluating the regulatory effects of exogenous insulin on glucose metabolism

**Figure 4:**
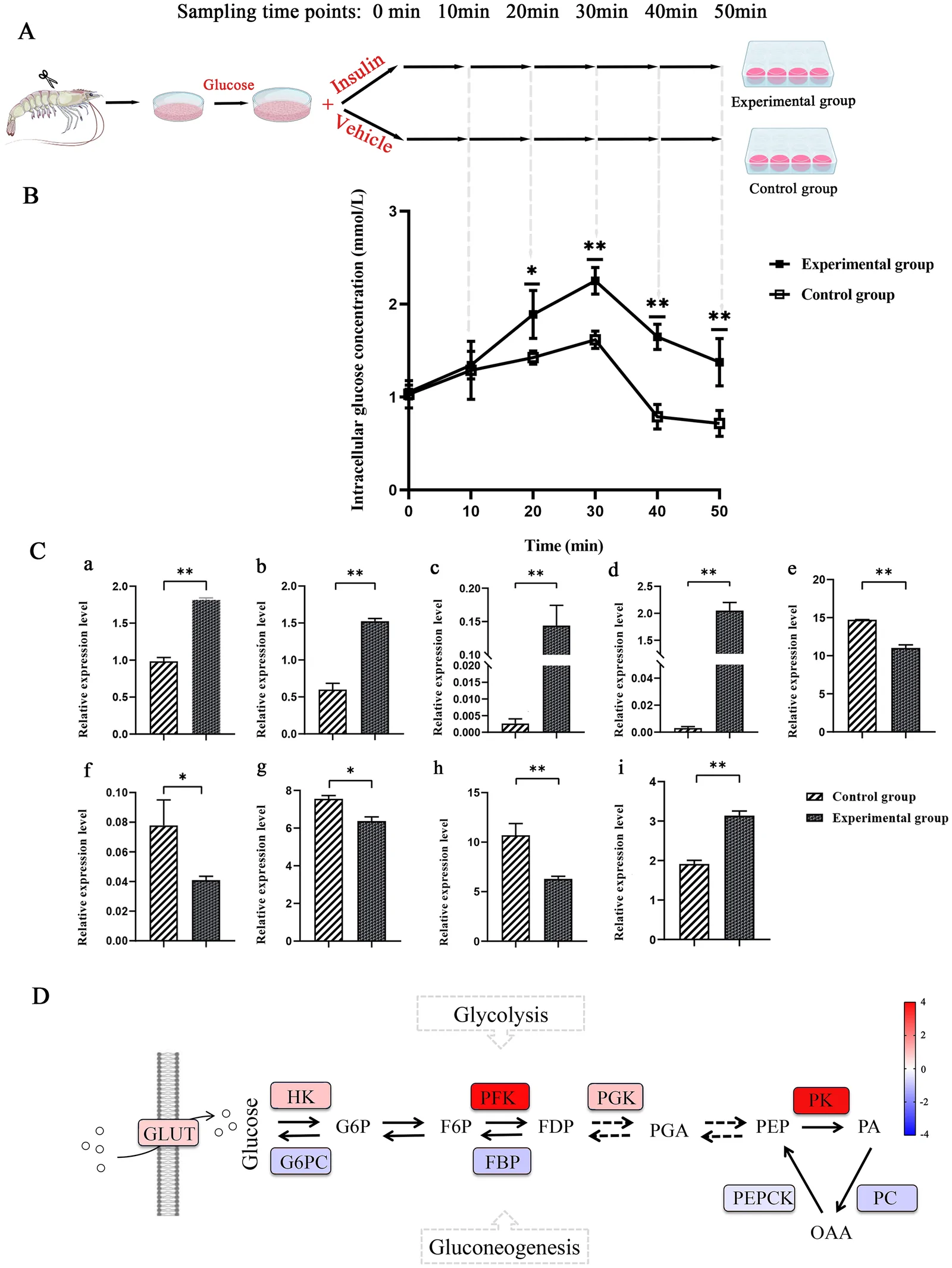
Effects of exogenous insulin on glucose metabolic responses in primary hepatopancreatic cells from *L. vannamei*. **(A)** Schematic diagram of glucose challenge and insulin treatment in primary hepatopancreatic cells. **(B)** Dynamic changes in intracellular glucose concentration from 0 to 50 min after insulin treatment. **(C)** Expression changes of glucose metabolism-related genes at 20 min after insulin treatment. **(a)** *HK*; **(b)** *PGK*; **(c)** *PK*; **(d)** *PFK*; **(e)** *PEPCK*; **(f)** *G6PC*; **(g)** *FBP*; **(h)** *PC*; **(i)** *GLUT*. **(D)** Schematic diagram of expression changes in glucose metabolism-related genes; the pathway structure is the same as that shown in **Figure 3D**. Red indicates up-regulated gene expression, and blue indicates down-regulated gene expression; color intensity represents the log_2_ fold change relative to the control group. Data are presented as the mean ± SD. *p < 0.05, **p < 0.01.

To determine whether the primary hepatopancreatic cell system could respond to exogenous hormonal stimulation, insulin treatment was performed following glucose challenge, and intracellular glucose concentration and glucose metabolism-related gene expression were monitored. As shown in **Figure 4A**, after glucose pretreatment, exogenous insulin was added to the treatment group, whereas an equal volume of solvent control was added to the control group. Samples were collected at 0, 10, 20, 30, 40, and 50 min for subsequent analyses.

Dynamic analysis of intracellular glucose concentration showed that both groups exhibited an initial increase followed by a decrease after glucose treatment; however, glucose accumulation was markedly enhanced in the insulin-treated group (**Figure 4B**). In the control group, intracellular glucose concentration peaked at approximately 30 min and then gradually declined. In contrast, the insulin-treated group showed significantly higher intracellular glucose levels after 20 min onward, reached a peak at 30 min at approximately 1.87-fold that of the control group, and remained relatively elevated at 40 and 50 min. These results suggest that exogenous insulin enhanced intracellular glucose accumulation and prolonged the elevated glucose state in primary hepatopancreatic cells.

To further assess the effects of exogenous insulin on glucose metabolism-related pathways, genes involved in glucose transport, glycolysis, and gluconeogenesis were examined at 20 min after insulin treatment. The expression levels of the glycolysis-related genes *HK*, *PGK*, *PK*, and *PFK* were significantly increased (**Figures 4C, a–d**), and *GLUT* was also significantly up-regulated (**Figures 4C, i**), indicating activation of glucose transport- and glycolysis-related transcriptional responses. In contrast, the gluconeogenesis-related genes *PEPCK*, *G6PC*, *FBP*, and *PC* were significantly down-regulated (**Figures 4C, e–h**). Overall, exogenous insulin elicited a coordinated glucose-metabolic response characterized by enhanced glucose transport, up-regulation of glycolysis-related genes, and down-regulation of gluconeogenesis-related genes (**Figure 4D**). These results demonstrate that the primary hepatopancreatic cell system can respond not only to exogenous glucose stimulation, but also to hormonal regulation by insulin, supporting its application for cellular-level assessment of insulin-mediated regulation of glucose metabolism.

### 3.5 Application of the primary hepatopancreatic cell system for modeling glucose metabolic responses under starvation-mimicking conditions *in vitro*

**Figure 5:**
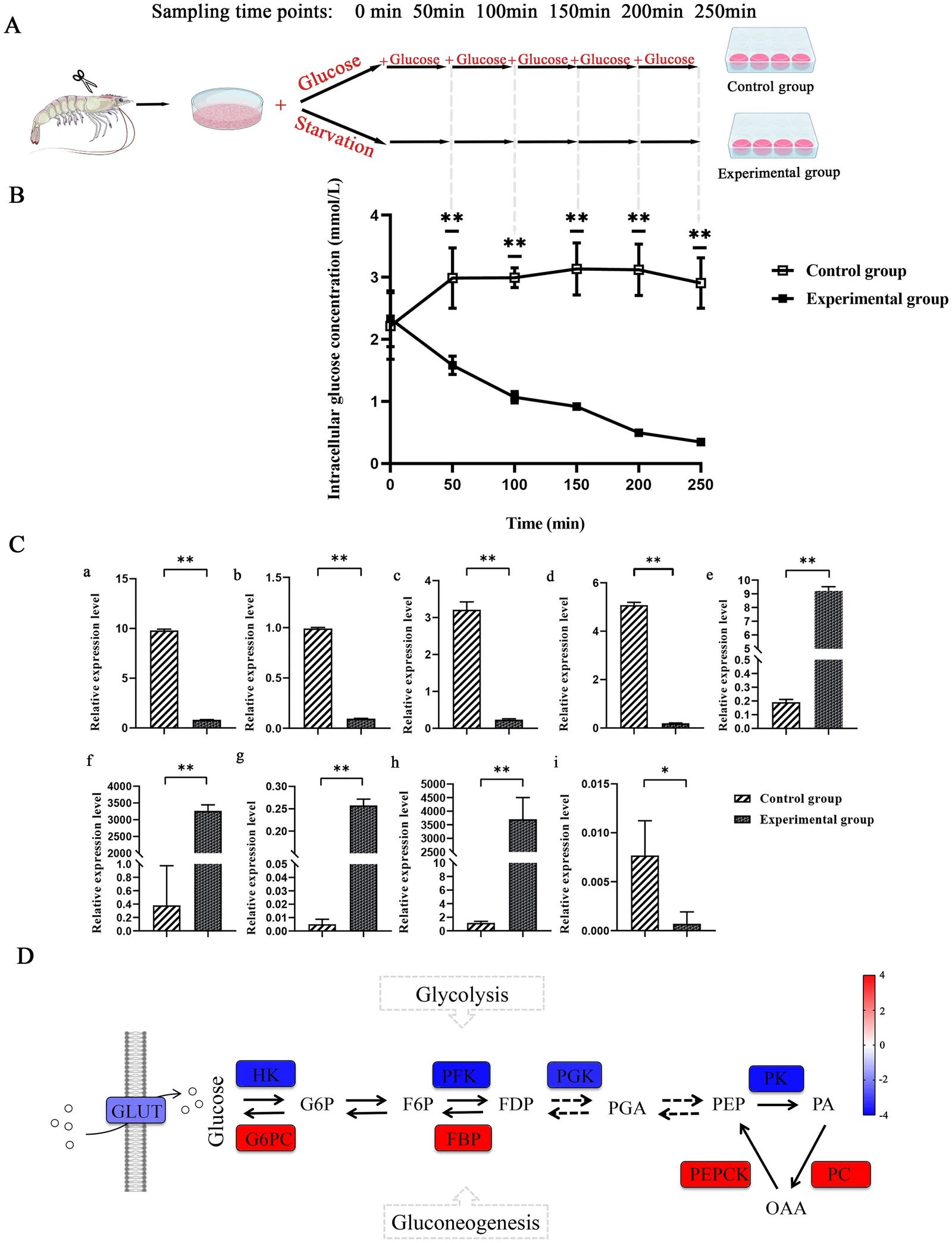
Glucose metabolic responses of primary hepatopancreatic cells from *L. vannamei* under starvation-mimicking conditions. **(A)** Schematic diagram of starvation-mimicking treatment and sampling in primary hepatopancreatic cells. The control group was supplemented with glucose solution every 50 min, whereas the treatment group was continuously cultured in glucose-free medium. **(B)** Dynamic changes in intracellular glucose concentration after starvation-mimicking treatment. **(C)** Expression changes of glucose metabolism-related genes at 200 min after starvation-mimicking treatment. **(a)** *HK*; **(b)** *PGK*; **(c)** *PK*; **(d)** *PFK*; **(e)** *PEPCK*; **(f)** *G6PC*; **(g)** *FBP*; **(h)** *PC*; **(i)** *GLUT*. **(D)** Schematic diagram of expression changes in glucose metabolism-related genes; the pathway structure is the same as that shown in **Figure 3D**. Red indicates up-regulated gene expression, and blue indicates down-regulated gene expression; color intensity represents the log_2_ fold change relative to the control group. Data are presented as the mean ± SD. *p < 0.05, **p < 0.01.

The primary hepatopancreatic cell system was further applied to an *in vitro* nutrient-deprivation model to evaluate its applicability under different metabolic conditions. As shown in **Figure 5A**, cells in the control group were supplemented with glucose solution every 50 min to maintain a continuous exogenous glucose supply, whereas cells in the starvation-mimicking group were maintained in glucose-free medium. Samples were collected at 0, 50, 100, 150, 200, and 250 min to measure intracellular glucose concentration and the expression of glucose metabolism-related genes.

Dynamic measurement of intracellular glucose concentration showed that the two groups had comparable glucose levels at the beginning of treatment. As the treatment progressed, intracellular glucose concentration in the control group remained relatively high and stabilized at approximately 3.0 mmol/L after 50 min. In contrast, intracellular glucose concentration in the starvation-mimicking group was significantly lower than that in the control group from 50 min onward and continued to decline over time, reaching a low level of approximately 0.4 mmol/L at 250 min (**Figure 5B**). These results indicate that restriction of exogenous glucose supply induced a sustained low-glucose state in primary hepatopancreatic cells, supporting the use of this system to model cellular starvation or nutrient deprivation *in vitro*.

Analysis of glucose metabolism-related gene expression showed that starvation-mimicking treatment induced a transcriptional response distinct from those observed after glucose challenge and insulin treatment (**Figure 5C**). At 200 min, the glycolysis-related genes *HK*, *PGK*, *PK*, and *PFK* were significantly down-regulated in the starvation-mimicking group compared with the control group (**Figures 5C, a–d**), indicating suppressed glycolysis-related transcription under low-glucose conditions. In contrast, the gluconeogenesis-related genes *PEPCK*, *G6PC*, *FBP*, and *PC* were significantly up-regulated (**Figures 5C, e–h**), while the glucose transporter gene *GLUT* was significantly down-regulated (**Figures 5C, i**). Together with the pathway schematic (**Figure 5D**), these results indicate that starvation-mimicking treatment elicited a coordinated metabolic response characterized by reduced glucose transport and glycolysis-related transcription, together with increased gluconeogenesis-related transcription. Overall, this primary hepatopancreatic cell system responded not only to glucose challenge and insulin stimulation, but also to nutrient deprivation, providing an *in vitro* model for studying metabolic adaptation in hepatopancreatic cells of *L. vannamei*.

### 3.6 Application of the primary hepatopancreatic cell system to pharmacological perturbation using linsitinib

**Figure 6:**
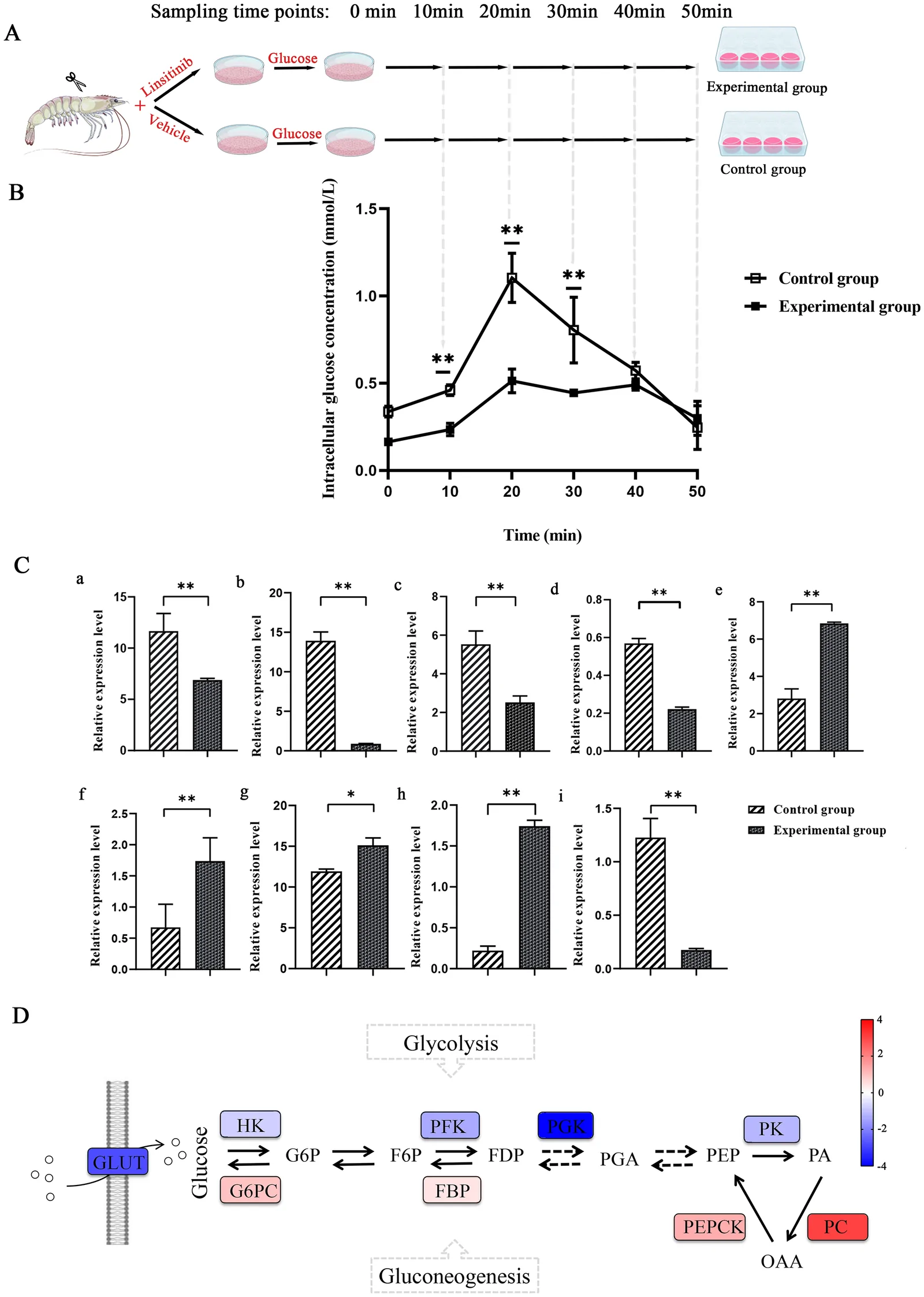
Glucose metabolic responses of primary hepatopancreatic cells from *L. vannamei* after linsitinib treatment. **(A)** Schematic diagram of linsitinib pretreatment followed by glucose challenge. **(B)** Dynamic changes in intracellular glucose concentration after glucose challenge following linsitinib pretreatment. **(C)** Expression changes in glucose metabolism-related genes at 20 min after glucose challenge. **(a)** *HK*; **(b)** *PGK*; **(c)** *PK*; **(d)** *PFK*; **(e)** *PEPCK*; **(f)** *G6PC*; **(g)** *FBP*; **(h)** *PC*; **(i)** *GLUT*. **(D)** Schematic diagram of expression changes in glucose metabolism-related genes; the pathway structure is the same as that shown in **Figure 3D**. Red indicates up-regulated gene expression, and blue indicates down-regulated gene expression; color intensity represents the log_2_ fold change relative to the control group. Data are presented as the mean ± SD. *p < 0.05, **p < 0.01.

To further evaluate whether this primary hepatopancreatic cell system could be used to assess glucose-metabolic responses under pharmacological perturbation, primary hepatopancreatic cells from *L. vannamei* were pretreated with linsitinib. Linsitinib, also known as OSI-906, is a small-molecule dual receptor tyrosine kinase inhibitor widely used to target IGF-1R and IR signaling in mammalian systems (31). As shown in **Figure 6A**, cells in the treatment group were pretreated with linsitinib, whereas those in the control group received an equal volume of solvent control. Both groups were then subjected to glucose challenge, and samples were collected at 0, 10, 20, 30, 40, and 50 min.

Dynamic measurement of intracellular glucose concentration showed that the control group exhibited a clear increase followed by a decrease after glucose treatment, with a peak at 20 min. In contrast, the glucose-induced increase in intracellular glucose was markedly attenuated in the linsitinib-treated group, in which intracellular glucose levels remained lower than those in the control group throughout the observation period and peaked at only approximately 0.5 mmol/L (**Figure 6B**). These results indicate that linsitinib treatment substantially weakened the glucose-responsive profile of primary hepatopancreatic cells.

Gene expression analysis further supported this result. Compared with the control group, linsitinib treatment down-regulated the glycolysis-related genes *HK*, *PGK*, *PK*, and *PFK* (**Figures 6C,a–d**) and significantly reduced the expression of the glucose transporter gene *GLUT* (**Figures 6C, i**). In contrast, the gluconeogenesis-related genes *PEPCK*, *G6PC*, *FBP*, and *PC* were generally up-regulated (**Figures 6C, e–h**). This expression pattern indicates that linsitinib treatment suppressed glucose transport- and glycolysis-related transcriptional responses while enhancing gluconeogenesis- related transcription. Together, these results show that the metabolic response of primary hepatopancreatic cells to glucose challenge can be effectively altered by linsitinib treatment, supporting the applicability of this system for evaluating glucose metabolic responses under pharmacological perturbation.

### 3.7 *In vivo* insulin-degrading enzyme knockdown combined with *in vitro* glucose challenge extends the functional validation capacity of the primary hepatopancreatic cell system

Because crustacean primary cells are highly sensitive to transfection, long-term culture, and exogenous reagent treatment (39), direct gene functional validation, such as RNAi, remains technically challenging at the primary cell level. To extend the application of the hepatopancreatic primary cell system to gene function studies, we adopted a strategy combining *in vivo* RNAi pretreatment with subsequent *in vitro* functional assays. In this workflow, target gene knockdown was first established at the whole-animal level, after which primary cells were isolated and subjected to glucose challenge under standardized *in vitro* conditions. This design allowed us to assess whether metabolic changes induced by *in vivo* gene perturbation could be captured in short-term primary cell assays.

**Figure 7:**
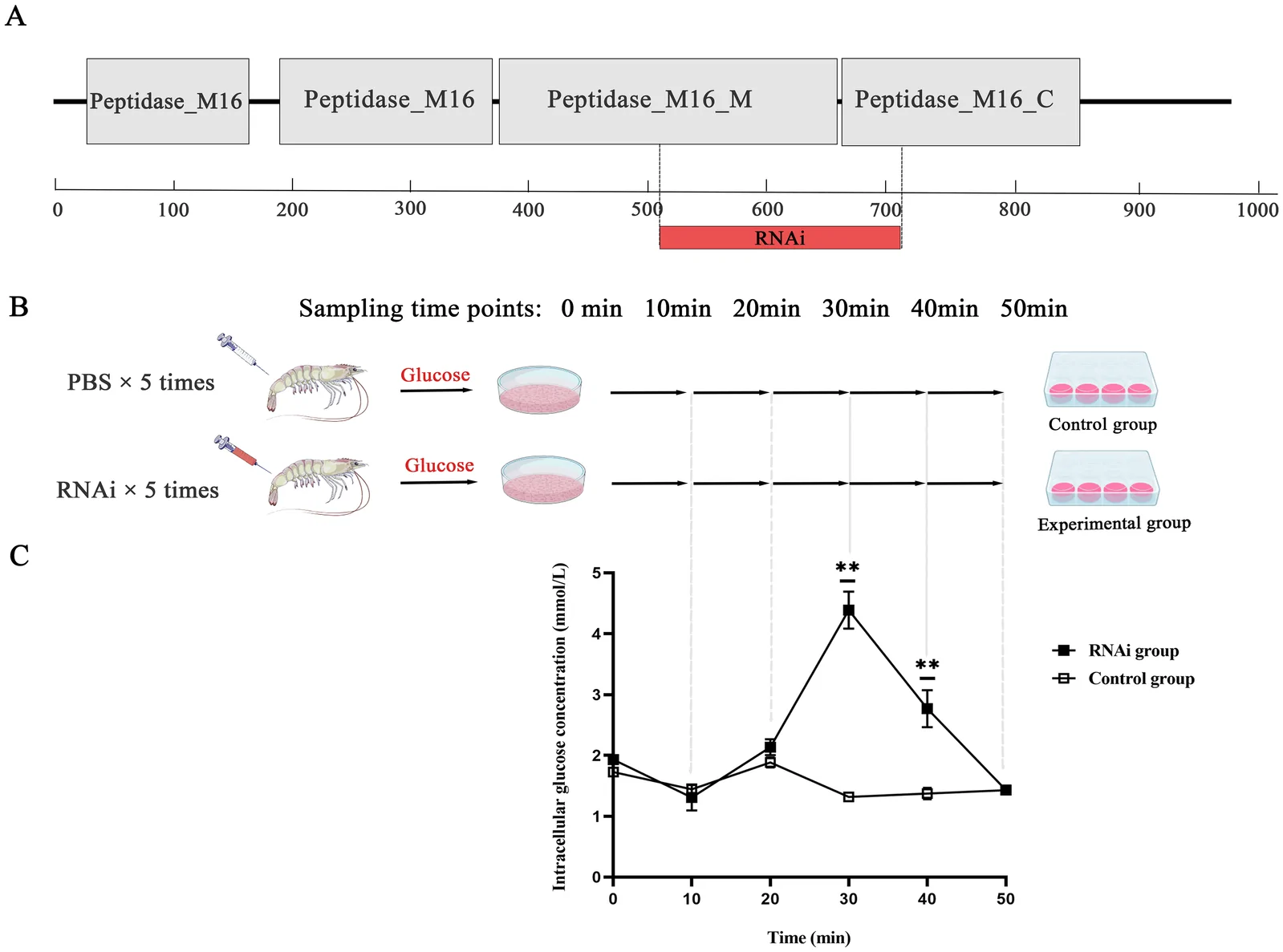
Changes in the glucose-challenge response of primary hepatopancreatic cells after *in vivo IDE* knockdown. **(A)** Schematic diagram of the IDE protein domains and RNAi target region. The red region indicates the RNAi target site. **(B)** Experimental workflow of *in vivo IDE* knockdown combined with primary hepatopancreatic cell isolation and subsequent *in vitro* glucose challenge. **(C)** Dynamic changes in intracellular glucose concentration in primary hepatopancreatic cells isolated from the PBS and dsIDE groups after glucose challenge. Data are presented as the mean ± SD. **p < 0.01.

The *IDE* gene was selected as the validation target. *IDE* is a highly conserved M16 family metalloprotease involved in insulin degradation and is closely associated with glucose homeostasis (40, 41). Based on transcriptomic data, a candidate *IDE* sequence was identified in *L. vannamei*. Domain analysis showed that the predicted protein contained conserved domains characteristic of the M16 metalloprotease family (**Figure 7A**). Accordingly, an RNAi target region was designed between the Peptidase_M16_M and Peptidase_M16_C domains for subsequent *in vivo* suppression (**Figure 7A**).

As shown in **Figures 7B, L***. vannamei* individuals were injected with PBS or dsIDE every 96 h for five consecutive treatments to establish a sustained *IDE* knockdown background (**Figure 7B**). After knockdown efficiency was confirmed by qRT-PCR, primary hepatopancreatic cells were isolated from the PBS and dsIDE groups and subjected to glucose challenge under the same culture conditions. Samples were collected at 0, 10, 20, 30, 40, and 50 min to examine dynamic changes in intracellular glucose concentration.

Primary hepatopancreatic cells derived from the dsIDE-treated group exhibited a stronger intracellular glucose accumulation response after glucose challenge (**Figure 7C**). Compared with the PBS control group, intracellular glucose concentration in the dsIDE group increased rapidly after treatment and reached a significantly higher peak at 30 min. At 40 min, intracellular glucose levels in the dsIDE group remained relatively elevated. Given the general role of *IDE* in the degradation and clearance of insulin or insulin-like peptides, reduced *IDE* expression may affect glucose regulation associated with insulin-like signaling. These results show that primary hepatopancreatic cells isolated after *in vivo IDE* knockdown retained detectable metabolic differences under *in vitro* glucose challenge conditions. Overall, the strategy combining *in vivo* RNAi, primary cell isolation, and *in vitro* functional testing provides a feasible approach to partially overcome the difficulty of direct transfection in crustacean primary cells.

### 3.8 Application of the primary hepatopancreatic cell system for evaluating delayed glucose responses after prolonged insulin exposure

We further evaluated whether the primary hepatopancreatic cell system could reflect changes in cellular glucose responsiveness after prolonged endocrine perturbation. Because prolonged insulin exposure has been used to alter insulin signaling and glucose responsiveness in mammalian cellular models (42, 43), an *in vivo* prolonged insulin exposure background was established by repeated injection of exogenous insulin. After treatment, primary hepatopancreatic cells were isolated and subjected to *in vitro* glucose challenge.

**Figure 8:**
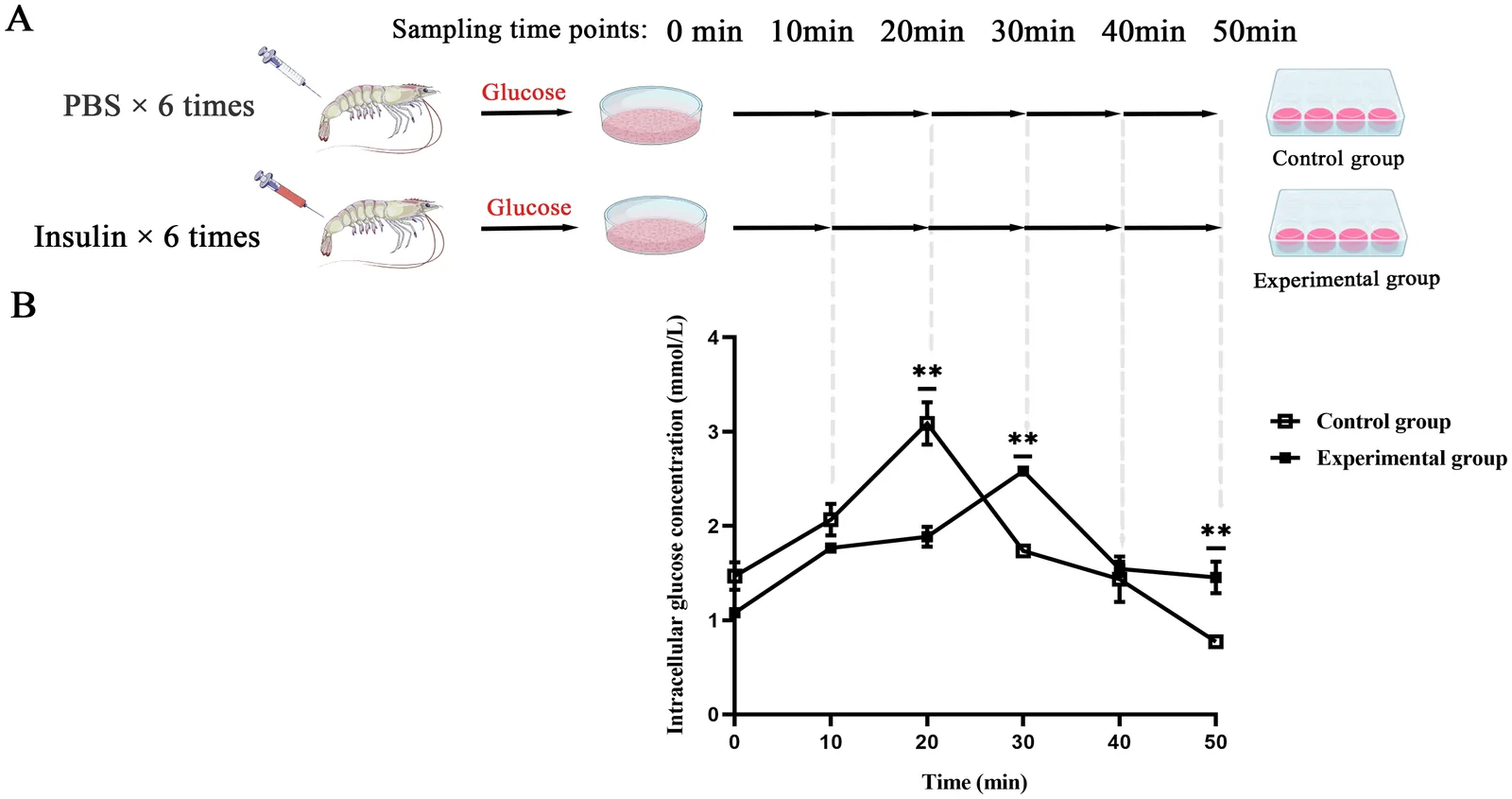
Glucose-challenge response of primary hepatopancreatic cells after prolonged exogenous insulin treatment. **(A)** Schematic diagram of prolonged exogenous insulin treatment combined with *in vitro* glucose challenge. **(B)** Dynamic changes in intracellular glucose concentration from 0 to 50 min after glucose challenge in primary hepatopancreatic cells derived from the PBS control and insulin-treated groups. Data are presented as the mean ± SD. **p < 0.01.

In this experiment, *L. vannamei* individuals were injected with PBS or exogenous insulin every 72 h for a total of six injections over 15 days. To minimize the acute effects of the final insulin injection on subsequent *in vitro* assays, hepatopancreatic tissues were collected 24 h after the last treatment. Primary hepatopancreatic cells were then isolated from the PBS control and prolonged insulin-treated groups and subjected to glucose challenge under the same *in vitro* conditions. Dynamic changes in intracellular glucose concentration were measured at 0, 10, 20, 30, 40, and 50 min (**Figure 8A**).

Primary hepatopancreatic cells derived from the PBS control group displayed a rapid and typical response, with intracellular glucose concentration increasing to a peak at approximately 20 min and then declining to a low level by 50 min (**Figure 8B**). In contrast, cells derived from the prolonged insulin-treated group showed a delayed glucose response, with the intracellular glucose peak shifted to approximately 30 min. Glucose levels also declined more slowly after the peak and remained relatively elevated during the subsequent observation period (**Figure 8B**). These results indicate that prolonged exogenous insulin exposure altered the temporal glucose-response profile of primary hepatopancreatic cells, as reflected by delayed glucose accumulation and a slower post-peak decline.

Overall, the delayed glucose response observed after prolonged insulin treatment suggests that this system can capture changes in the glucose metabolic status of hepatopancreatic cells following sustained endocrine perturbation, providing an expandable *in vitro* validation strategy for studies of insulin-like endocrine regulation in crustaceans.

### 3.9 Application of the primary hepatopancreatic cell system for cytochemical observation of glycogen and lipid storage

To further expand the application of the primary hepatopancreatic cell system from *L. vannamei* in metabolic status assessment, an optimized cell-smear preparation and cytochemical staining procedure was used. PAS and ORO staining were performed to visualize glycogen- and lipid-storage characteristics, respectively, in primary hepatopancreatic cells under different nutritional states. PAS-positive signals typically appear as magenta deposits, whereas ORO-positive lipid droplets appear orange-red. Staining intensity was further evaluated semi-quantitatively using average optical density (AOD), calculated as the integrated optical density divided by the corresponding positive-stained area.

**Figure 9:**
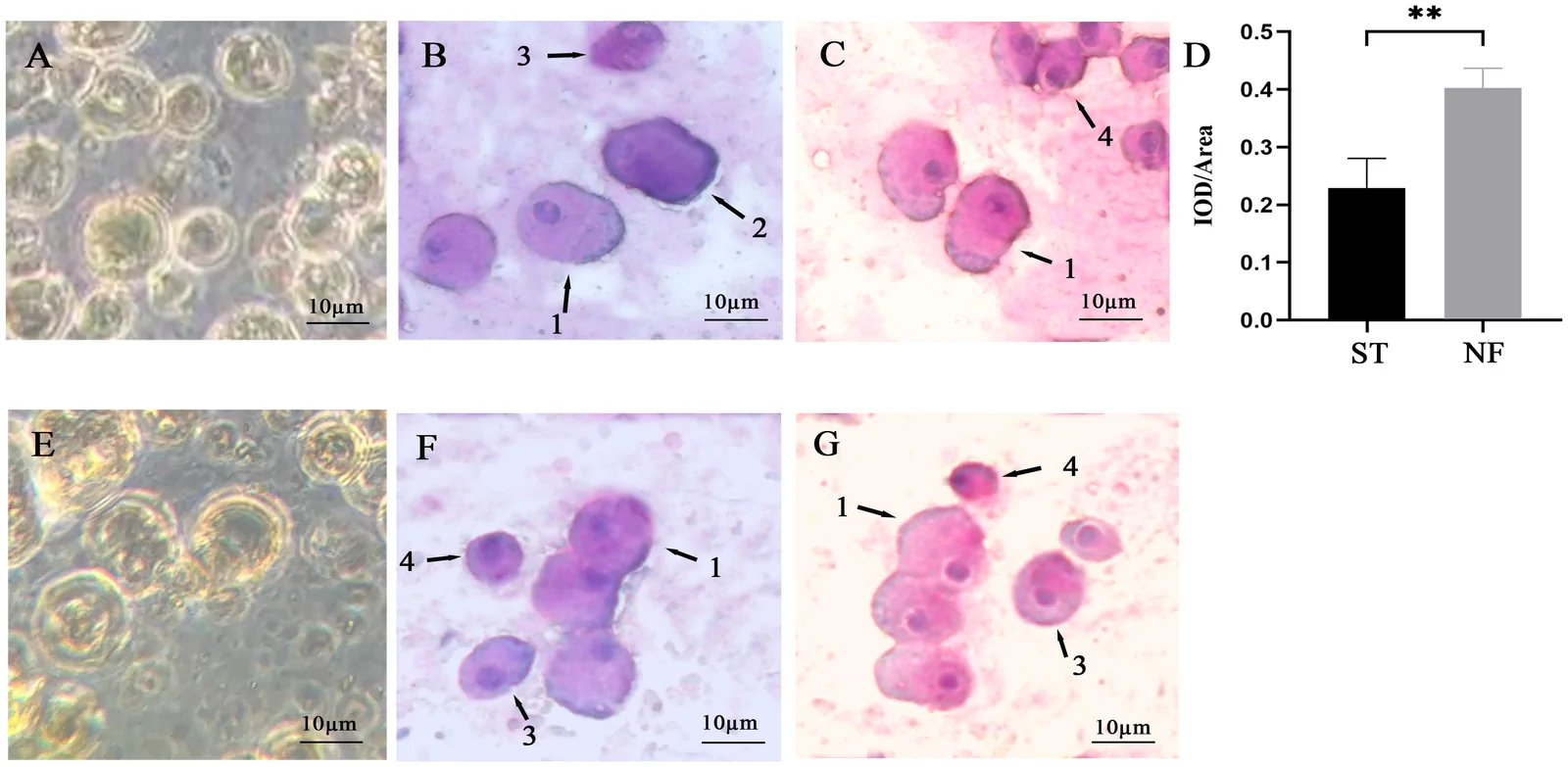
Periodic acid–Schiff (PAS) staining of primary hepatopancreatic cells from *L. vannamei* under different nutritional conditions. **(A, E)** Morphological observation of primary hepatopancreatic cells before staining. **(B, F)** PAS staining of cells from the starvation-treated group. **(C, G)** PAS staining of cells from the normally fed group. Magenta deposits indicate PAS-positive signals. Numbers 1–4 indicate representative putative B-like, R-like, F-like, and E-like cells, respectively. **(D)** Quantitative analysis of PAS-positive staining intensity, expressed as average optical density (AOD). ST, starvation-treated group; NF, normally fed group. Data are presented as the mean ± SD. **p < 0.01. Scale bar = 10 μm.

PAS staining revealed clear differences in glycogen-associated positive signals between the two nutritional conditions (**Figure 9**). Cells from the starvation-treated group displayed weaker PAS-positive signals and reduced magenta deposition compared with those from the normally fed group (**Figures 9B, C, F, G**). Consistent with the morphological observations, quantitative image analysis showed that the AOD of PAS-positive staining was significantly lower in the starvation-treated group than in the normally fed group (**Figure 9D**). These results indicate that PAS staining can reflect differences in the glycogen storage status of primary hepatopancreatic cells under different nutritional conditions.

**Figure 10:**
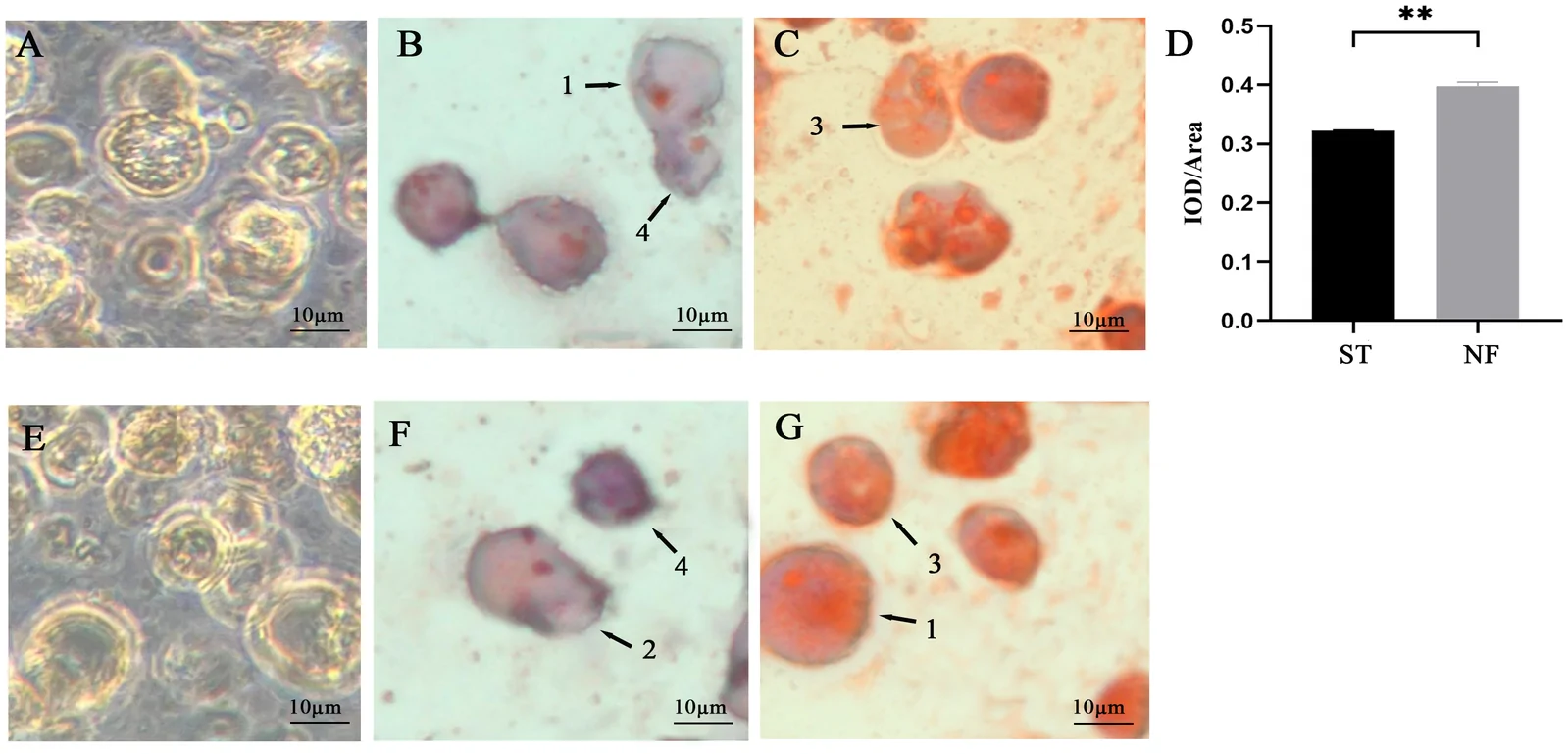
Oil Red O (ORO) staining of primary hepatopancreatic cells from *L. vannamei* under different nutritional conditions. **(A, E)** Morphological observation of primary hepatopancreatic cells. **(B, F)** ORO staining of cells from the starvation-treated group. **(C, G)** ORO staining of cells from the normally fed group. Orange-red lipid droplets indicate ORO-positive signals. Numbers 1–4 indicate representative putative B-like, R-like, F-like, and E-like cells, respectively. **(D)** Quantitative analysis of ORO-positive staining intensity, expressed as average optical density (AOD). ST, starvation-treated group; NF, normally fed group. Data are presented as the mean ± SD. **p < 0.01. Scale bar = 10 μm.

ORO staining further showed that intracellular lipid-storage status also varied with nutritional condition (**Figure 10**). Compared with the normally fed group, cells from the starvation-treated group showed weaker orange-red lipid droplet signals, with reduced lipid droplet abundance and staining intensity (**Figures 10B, F**). In contrast, more evident ORO-positive signals were observed in cells from the normally fed group (**Figures 10C, G**). Quantitative analysis confirmed that the AOD of ORO-positive staining was significantly lower in the starvation-treated group than in the normally fed group (**Figure 10D**). These results indicate that the primary hepatopancreatic cell system can be used to visualize changes in lipid-storage status.

Taken together, PAS and ORO staining showed that primary hepatopancreatic cells from *L. vannamei* displayed observable differences in glycogen and lipid storage under different nutritional states. In addition, PAS-positive signals and ORO-positive lipid droplets were observed in cells belonging to several putative morphological groups, indicating that the optimized staining procedure allowed cytochemical and morphological features to be observed simultaneously.

## 4 Discussion

### 4.1 A primary hepatopancreatic cell system provides a tractable *in vitro* platform for crustacean endocrine and metabolic research

The long-standing lack of robust cellular tools has been a major constraint on mechanistic studies of crustacean cell biology, endocrine regulation, and metabolic control (39). Unlike vertebrates and insects, crustaceans still lack well-established continuous cell lines that can be stably passaged and broadly applied (44, 45). Previous reviews have identified several persistent barriers to crustacean and marine invertebrate cell culture, including suboptimal culture media, limited maintenance of stemness and proliferative competence, insufficient omics-guided optimization, and unclear criteria for cell-type selection (44, 46). In this context, primary cell systems should not be regarded merely as provisional substitutes for continuous cell lines. Instead, they provide a practical and physiologically relevant approach for cell-level functional studies in crustaceans, particularly in endocrine and metabolic research.

Previous studies have demonstrated the feasibility of shrimp primary cells for *in vitro* applications, although most have focused on pathogen-infection models (44, 47). For example, primary cultures derived from the lymphoid organ, hemocytes, or hepatopancreas of *M. japonicus*, *Fenneropenaeus chinensis*, *Penaeus monodon*, and *L. vannamei* have been used to investigate viral infection, cytopathic effects, viral replication, and host immune responses (48–50). By comparison, primary cell systems designed for nutritional metabolism, hormonal stimulation, and endocrine signaling remain insufficiently developed in shrimp.

In the present study, the hepatopancreas was selected for primary cell model establishment because of its central role in crustacean nutrient processing and metabolic regulation. As the principal metabolic organ in decapod crustaceans, the hepatopancreas integrates digestive enzyme secretion, nutrient absorption, energy storage, nutrient transport, and metabolic redistribution, thereby linking nutritional status, endocrine regulation, and energy homeostasis (19, 51). Classical histological studies have shown that the crustacean hepatopancreatic epithelium is mainly composed of E, F, R, and B cells, which are morphologically and functionally distinct (18, 51, 52). E cells are relatively undifferentiated and are generally considered proliferative progenitor cells within the tubular germinal zone. F cells are primarily involved in the synthesis and secretion of digestive enzymes, whereas R cells contribute to nutrient absorption and metabolism, lipid and glycogen storage, and detoxification (18). B cells are characterized by prominent vacuolar structures and have traditionally been associated with intracellular digestion and assimilation of endocytosed materials (18, 51). These specialized cell types together provide the cellular basis for the digestive, absorptive, and metabolic regulatory functions of the crustacean hepatopancreas (18). Consistent with this cellular architecture, the isolated cell preparation contained several morphologically distinct populations that were tentatively classified as B-like, R-like, F-like, and E-like cells. These assignments were based solely on light-microscopic features and were not validated using cell type-specific molecular markers; they should therefore be regarded as preliminary morphology-based classifications rather than definitive cell-type identifications. Recent single-cell and single-nucleus transcriptomic studies of the *L. vannamei* hepatopancreas have resolved multiple transcriptionally distinct cell populations and identified candidate markers for future cell-type characterization (53, 54). These datasets provide an important basis for linking classical morphological phenotypes with molecularly defined cell populations in future studies.

Importantly, the objective of the present study was not to establish purified cultures of individual hepatopancreatic cell types, but rather to develop a short-term primary cell system in which freshly isolated hepatopancreatic cells could be examined shortly after isolation. This design was intended to minimize culture-associated alterations while preserving, as far as possible, the heterogeneous cellular composition and morphological diversity of the original cell preparation. The resulting mixed-cell system therefore provides a population-level functional readout under controlled *in vitro* conditions and enables comparison with metabolic and endocrine responses previously observed in whole-animal studies. Accordingly, the functional results obtained here should be interpreted as aggregate responses of the heterogeneous primary cell population rather than as cell type-specific effects.

Building on existing medium formulations, we further optimized culture conditions by evaluating the effects of pH, NaCl supplementation, FBS content, and SME supplementation on cell viability. Among the tested conditions, pH 7.3, 4‰ NaCl, 15% FBS, and 3% SME were more favorable for short-term cell maintenance. Taken together, the primary hepatopancreatic cell system established here provides a viable short-term culture model that maintains cell viability and morphological heterogeneity under optimized conditions, thereby offering a practical platform for investigating integrated endocrine and metabolic responses in shrimp.

### 4.2 Primary hepatopancreatic cells enable *in vitro* validation of representative metabolic and endocrine responses

The significance of this study extends beyond the establishment of a short-term primary hepatopancreatic cell system from *L. vannamei*. More importantly, this system responded to representative nutritional, endocrine, and pharmacological challenges with glucose-metabolic patterns consistent with their respective physiological contexts. Glucose loading, exogenous insulin treatment, starvation-mimicking conditions, and linsitinib exposure modeled distinct experimental contexts, including nutrient input, hormonal stimulation, nutrient deprivation, and linsitinib-mediated pharmacological perturbation, respectively. Across these conditions, primary hepatopancreatic cells exhibited measurable intracellular glucose dynamics, together with coordinated transcriptional changes in genes involved in glucose transport, glycolysis, and gluconeogenesis. These findings indicate that the system not only maintains a basal cellular state *in vitro*, but also provides a workable platform for cell-level validation of glucose metabolism and endocrine-metabolic regulation in crustaceans.

In the glucose-loading assay, primary hepatopancreatic cells rapidly sensed exogenous glucose and displayed a dynamic intracellular glucose profile characterized by a transient increase followed by a decline. Consistently, glucose treatment up-regulated *GLUT* and glycolysis-related genes, including *HK*, *PGK*, and *PFK*, while generally down-regulating gluconeogenesis-related genes such as *PEPCK*, *G6PC*, *FBP*, and *PC*. This pattern suggests that, under glucose-replete conditions, the cells enhance glucose transport and glycolytic utilization while limiting transcriptional programs associated with endogenous glucose production. It is also broadly consistent with glucose clearance and metabolic readjustment observed after *in vivo* glucose loading in crustaceans (8, 55). Notably, previous *in vivo* glucose-loading studies in *L. vannamei* usually required repeated sampling from large numbers of shrimp over several hours (5, 26), whereas the present primary cell system captured intracellular glucose dynamics within approximately 50 min. This short response window makes the system suitable for rapid screening, early-response analysis, and cell-level functional validation under glucose stimulation.

Exogenous insulin treatment further confirmed the endocrine responsiveness of this system. When insulin was added after glucose loading, primary hepatopancreatic cells showed greater intracellular glucose accumulation, accompanied by increased expression of glucose transport- and glycolysis-related genes and reduced expression of gluconeogenic genes. These results indicate that insulin shifted the cellular response to glucose loading toward an enhanced glucose-utilization state. Previous studies have shown that ILPs and their signaling components participate in the regulation of glucose homeostasis, growth, and energy metabolism in crustaceans (56). In *L. vannamei*, ILPs have also been implicated in maintaining hemolymph glucose homeostasis and regulating glucose metabolic processes (5, 27). Therefore, the present findings support the use of primary hepatopancreatic cells as an *in vitro* model for examining insulin-like endocrine regulation of glucose metabolism in crustaceans.

In contrast, starvation-mimicking conditions induced a continuous decline in intracellular glucose levels, accompanied by down-regulation of glycolysis-related genes, up-regulation of gluconeogenesis-related genes, and reduced *GLUT* expression. These changes indicate that, under limited exogenous glucose availability, primary hepatopancreatic cells can mount a transcriptional response consistent with nutrient deprivation. In crustaceans, starvation generally triggers energy mobilization, glycogen depletion, and metabolic pathway redistribution, with the hepatopancreas serving as the principal organ coordinating these processes (57). The present *in vitro* system reproduced the directional changes in glucose metabolism-related genes under nutrient-deprived conditions, suggesting that it can model short-term cellular responses to glucose limitation in a more controllable environment than whole-animal starvation experiments.

Linsitinib treatment further extended the applicability of this system to pharmacological perturbation studies. As a dual receptor tyrosine kinase inhibitor targeting IGF-1R and IR, linsitinib is widely used to interfere with insulin-like signaling-related processes (30, 58). In the present study, linsitinib attenuated the dynamic response of primary hepatopancreatic cells to glucose loading, down-regulated genes associated with glycolysis and glucose transport, and up-regulated gluconeogenesis-related genes. These changes are broadly consistent with mammalian studies showing that linsitinib inhibits IGF-1R/IR signaling and affects glucose metabolism in a similar direction (49, 59). Thus, the primary hepatopancreatic cell system can capture glucose-metabolic changes associated with linsitinib treatment and can be used for cell-level assessment of metabolic responses under pharmacological intervention.

Previous whole-animal studies across several crustacean species, including *L. vannamei*, *Macrobrachium rosenbergii*, *Marsupenaeus japonicus*, and several crab species, have revealed broadly comparable physiological patterns of glucose regulation (7, 29, 60–62). Glucose loading induces dynamic changes in circulating glucose accompanied by adjustments in glucose transport, glycolysis, and gluconeogenesis (7, 60, 63), whereas insulin or ILP stimulation promotes glucose utilization and regulates key glucose-metabolic genes (5, 7). Conversely, starvation induces mobilization of carbohydrate reserves and metabolic reprogramming (5, 7), while genetic or endocrine perturbation of glucose-regulatory signaling alters glucose homeostasis and associated metabolic pathways (7, 26, 60, 61). Importantly, the primary hepatopancreatic cells examined here exhibited broadly consistent directional changes in intracellular glucose dynamics and metabolism-related gene expression. This cross-level consistency supports the physiological relevance of the present cell system and indicates that it can capture key aspects of *in vivo* metabolic responses under controlled *in vitro* conditions, thereby providing a useful cellular complement to whole-animal studies.

Collectively, these findings demonstrate that primary hepatopancreatic cells from *L. vannamei* retain representative nutrient- and endocrine-responsive metabolic properties *in vitro*. Together with their short response window, controllable culture conditions, and flexible treatment options, these features support the use of this system as an accessible cell-level platform for investigating nutrient sensing, insulin-like endocrine regulation, and glucose metabolic homeostasis in crustaceans.

### 4.3 *In vivo* perturbation combined with *in vitro* primary cell assays expands the functional validation capacity of the hepatopancreatic cell system

Beyond short-term *in vitro* stimulation assays, this study further developed an experimental framework that integrates *in vivo* perturbation with subsequent *in vitro* functional assessment of primary hepatopancreatic cells. This approach was designed to address a major practical limitation of crustacean primary cell systems: isolated primary cells are highly vulnerable to transfection reagents, prolonged culture, and intensive exogenous treatments (47). Direct transfection-mediated RNAi, long-term hormonal exposure, or sustained pharmacological intervention *in vitro* can rapidly impair cell integrity and viability, often resulting in extensive cell death. Establishing gene- or endocrine-state perturbations at the whole-animal level, followed by primary cell isolation within the effective perturbation window, therefore provides a more tractable and biologically reliable route for functional analysis under standardized *in vitro* conditions.

This strategy was first tested using *IDE* gene interference. *IDE* expression was suppressed *in vivo* in *L. vannamei*, after which primary hepatopancreatic cells were isolated and subjected to an *in vitro* glucose-loading assay. Cells from the dsIDE group showed a stronger intracellular glucose accumulation response, with significantly higher glucose levels than those of the PBS control group at 30 and 40 min. Given the role of *IDE* in degrading and clearing insulin and related peptide hormones, this response is consistent with enhanced insulin-like regulatory activity after *IDE* suppression (40, 64). Importantly, these results indicate that the metabolic state induced by *in vivo IDE* interference can be partially retained after short-term cell isolation and detected through an *in vitro* functional assay. Thus, the primary hepatopancreatic cell system can serve not only as a model for short-term exogenous stimulation, but also as a cellular readout platform for functional states induced by *in vivo* gene perturbation.

Long-term exogenous insulin treatment further extended the applicability of this framework. Unlike acute *in vitro* insulin stimulation, prolonged *in vivo* insulin exposure represents a sustained endocrine perturbation that is difficult to reproduce reliably in crustacean primary cells under direct culture conditions (46, 47). In mammalian systems, chronic hyperinsulinemic exposure can alter insulin responsiveness and glucose metabolism, often accompanied by changes in glucose uptake and insulin signaling (43, 65). Based on this rationale, primary hepatopancreatic cells were isolated after long-term *in vivo* insulin treatment and examined under standardized *in vitro* glucose-loading conditions. The results showed that cells derived from the PBS group responded rapidly to glucose loading, with intracellular glucose levels peaking at approximately 20 min, whereas the glucose peak in cells from the long-term insulin-treated group was delayed to approximately 30 min. This delayed response indicates that prolonged exogenous insulin exposure altered the temporal glucose-response profile of hepatopancreatic cells. Notably, this phenomenon is, to some extent, consistent with the rationale and phenotypic tendency of mammalian models in which chronic hyperinsulinemic exposure induces reduced insulin sensitivity (66).

Taken together, the *IDE* interference and long-term insulin treatment experiments demonstrate that the primary hepatopancreatic cell system can be extended from a short-term *in vitro* stimulation model to a functional validation platform for *in vivo* gene and endocrine perturbations. By combining the physiological relevance of whole-animal manipulation with the controllability of primary cell assays, this approach is particularly suitable for crustacean studies in which primary cells are difficult to transfect, poorly suited to prolonged culture, and not supported by stable continuous cell lines. Therefore, *in vivo* perturbation followed by *in vitro* functional readout provides a useful technical route for dissecting glucose metabolic regulation, insulin-like signaling, and nutrition-related metabolic phenotypes in shrimp.

### 4.4 Cytochemical staining provides complementary phenotypic readouts of energy-storage status in primary hepatopancreatic cells

In addition to monitoring intracellular glucose dynamics and assessing the expression of glucose metabolism-related genes, this study further used PAS and ORO staining to evaluate the energy-storage phenotype of primary hepatopancreatic cells at the cytochemical level. PAS staining detects glycogen and other polysaccharide-rich structures (67), whereas ORO staining visualizes intracellular neutral lipids and lipid droplets (68). Compared with cells from the normally fed group, primary hepatopancreatic cells from the starvation-treated group displayed weaker PAS-positive signals and reduced ORO-positive lipid droplet staining, indicating decreased glycogen and neutral lipid storage under nutrient-deprived conditions. These cytochemical changes are consistent with the decreased intracellular glucose levels, down-regulation of glycolysis-related genes, and up-regulation of gluconeogenesis-related genes observed in the starvation- mimicking assay. Thus, PAS and ORO staining provide complementary phenotypic evidence that the primary hepatopancreatic cell system can reflect changes in cellular energy storage under different nutritional states.

From a platform perspective, cytochemical staining adds an intuitive morphological layer to the assessment of hepatopancreatic metabolic status. Unlike glucose-content measurements and gene-expression analyses, PAS and ORO staining directly visualize intracellular carbohydrate and lipid reserves, thereby linking metabolic readouts to cellular phenotypes. Notably, after staining, several putative morphological groups remained distinguishable based on their light microscopic features, indicating that the optimized cell smear and staining workflow preserved sufficient cellular architecture for parallel cytochemical and morphological observation. Future studies should combine candidate markers for specific hepatopancreatic cell populations with *in situ* hybridization, immunological validation, flow cytometric sorting, and single cell transcriptomic approaches to further define cell identities and population composition.

## 5 Conclusion

In this study, a primary hepatopancreatic cell culture system from *L. vannamei* was established and optimized. The isolated cells retained typical morphological features of hepatopancreatic cell populations, and culture conditions favorable for short-term cell maintenance were identified. Functional validation demonstrated that this system generated glucose-metabolic responses consistent with the expected biological effects of glucose loading, exogenous insulin treatment, starvation mimicking, and linsitinib-mediated pharmacological perturbation, as reflected by dynamic changes in intracellular glucose levels and coordinated regulation of glucose metabolism-related genes. Furthermore, experiments combining *in vivo IDE* interference or long-term exogenous insulin exposure with subsequent *in vitro* glucose-loading assays showed that this system can support cellular functional readouts under both gene perturbation and sustained endocrine disturbance. With an improved cell-smear and cytochemical staining workflow, PAS and ORO staining further enabled visualization of energy-storage phenotypes in primary hepatopancreatic cells. Overall, this study establishes a tractable primary hepatopancreatic cell platform for investigating glucose metabolism and insulin-like endocrine regulation in crustaceans, providing a useful experimental tool for dissecting nutrient–endocrine–metabolic interactions at the cellular level.

## Data Availability

The datasets presented in this study can be found in online repositories. The names of the repository/repositories and accession number(s) can be found in the article/supplementary material.

## References

[1] FAO . The State of World Fisheries and Aquaculture 2024: Blue Transformation in Action. Rome: FAO (2024). doi: 10.4060/cd0683en

[2] Barua H Acharjee MR Giteru SG Chowdhury M Wu H Kumar L . Dietary phospholipids and their impact on crustacean physiology: growth, metabolism, immunity, and beyond. Aquac Nutr. (2025) 2025:8180797. doi: 10.1155/anu/8180797 40547201 PMC12181668

[3] Webster SG Keller R Dircksen H . The CHH-superfamily of multifunctional peptide hormones controlling crustacean metabolism, osmoregulation, moulting, and reproduction. Gen Comp Endocrinol. (2012) 175:217–33. doi: 10.1016/j.ygcen.2011.11.035 22146796

[4] Chen Y Chi S Zhang S Dong X Yang Q Liu H . Effect of black soldier fly (Hermetia illucens) larvae meal on lipid and glucose metabolism of Pacific white shrimp Litopenaeus vannamei. Br J Nutr. (2022) 128:1674–88. doi: 10.1017/s0007114521004670 34814963

[5] Gutiérrez A Nieto J Pozo F Stern S Schoofs L . Effect of insulin/IGF-I like peptides on glucose metabolism in the white shrimp Penaeus vannamei. Gen Comp Endocrinol. (2007) 153:170–5. doi: 10.1016/j.ygcen.2007.04.014 17574553

[6] Montiel-Arzate A Sánchez-Castrejón E Camacho-Jiménez L Díaz F Ponce-Rivas E . Effect of recombinant crustacean hyperglycemic hormones rCHH-B1 and rCHH-B2 on lipid metabolism in the Pacific white shrimp Litopenaeus vannamei. Aquac Res. (2020) 51:4267–78. doi: 10.1111/are.14769 40046247

[7] Su M Zhang X Yuan J Zhang X Li F . The role of insulin-like peptide in maintaining hemolymph glucose homeostasis in the Pacific white shrimp Litopenaeus vannamei. Int J Mol Sci. (2022) 23:3268. doi: 10.3390/ijms23063268 35328689 PMC8948857

[8] Zhang X Pan L Tong R Li Y Si L Chen Y . Effects of crustacean hyperglycaemic hormone RNA interference on regulation of glucose metabolism in Litopenaeus vannamei after ammonia-nitrogen exposure. Br J Nutr. (2022) 127:823–36. doi: 10.1017/S0007114521001574 33988091

[9] Jiang Q Gao W Xu W Chen A Ding Q Gao X . Postprandial regulation of glucose metabolism and insulin signaling pathways in Macrobrachium rosenbergii. Aquac Rep. (2024) 39:102501. doi: 10.1016/j.aqrep.2024.102501 42574925

[10] Ma J Zeng L Lu Y . Penaeid shrimp cell culture and its applications. Rev Aquac. (2017) 9:88–98. doi: 10.1111/raq.12106 40046247

[11] Li W Nguyen VT Corteel M Dantas-Lima JJ Van Thuong K Van Tuan V . Characterization of a primary cell culture from lymphoid organ of Litopenaeus vannamei and use for studies on WSSV replication. Aquaculture. (2014) 433:157–63. doi: 10.1016/j.aquaculture.2014.05.046 42574925

[12] Sudarshan G Weil S Jasińska W Manor R Goldstein O Aflalo ED . Correlation between metabolomic profile and proliferation of Macrobrachium rosenbergii primary embryonic cell culture. Front Mar Sci. (2023) 10:1270491. doi: 10.3389/fmars.2023.1270491

[13] Li W Vo Van T Thuong K Bossier P Hans Nauwynck . Eye extract improves cell migration out of lymphoid organ explants of L. vannamei and viability of the primary cell cultures. In Vitro Cell Dev Biol Anim. (2015) 51:651–4. doi: 10.1007/s11626-015-9882-2 25792199

[14] Sivakumar S Swaminathan TR Anandan R Kalaimani N . Medium optimization and characterization of cell culture system from Penaeus vannamei for adaptation of white spot syndrome virus (WSSV). J Virol Methods. (2019) 270:38–45. doi: 10.1016/j.jviromet.2019.04.016 31009654

[15] Chen SN Wang CS . Establishment of cell culture systems from penaeid shrimp and their susceptibility to white spot disease and yellow head viruses. Methods Cell Sci. (1999) 21:199–206. doi: 10.1023/a:1009885929335 10627672

[16] Zhao Y Guo L Guo H . Routine development of long-term primary cell culture and finite cell line from the hemolymph of greasyback shrimp (Metapenaeus ensis) and virus susceptibility. Aquaculture. (2023) 563:739007. doi: 10.1016/j.aquaculture.2022.739007 42574925

[17] Han Q Li P Lu X Guo Z Guo H . Improved primary cell culture and subculture of lymphoid organs of the greasyback shrimp Metapenaeus ensis. Aquaculture. (2013) 410-411:101–13. doi: 10.1016/j.aquaculture.2013.06.024 42574925

[18] Vogt G . Functional cytology of the hepatopancreas of decapod crustaceans. J Morphol. (2019) 280:1405–44. doi: 10.1002/jmor.21040 31298794

[19] Castejón D Rotllant G Alba-Tercedor J Font-i-Furnols M Ribes E Durfort M . Morphology and ultrastructure of the midgut gland (hepatopancreas) during ontogeny in the common spider crab Maja brachydactyla Balss, 1922 (Brachyura, Majidae). Arthropod Struct Dev. (2019) 49:137–51. doi: 10.1016/j.asd.2018.11.013 30557625

[20] Cervellione F McGurk C Eriksen TB Van den Broeck W . Use of computer-assisted image analysis for semi-quantitative histology of the hepatopancreas in whiteleg shrimp Penaeus vannamei (Boone). J Fish Dis. (2016) 40:1223–34. doi: 10.1111/jfd.12599 28032356

[21] Arumugam U Prabhakar T Koteeswaran A Ravikumar G . Establishment of primary cell culture from hepatopancreas of Penaeus monodon for the study of white spot syndrome virus (WSSV). Asian Fish Sci. (2002) 15:365–70. doi: 10.33997/j.afs.2002.15.4.008

[22] Wei Z Zhuang Y Liu X Zou D Mai K Sun Z . Leucine promotes protein synthesis of juvenile white shrimp Litopenaeus vannamei through TOR signaling pathway. Aquaculture. (2023) 564:739060. doi: 10.1016/j.aquaculture.2022.739060 42574925

[23] Luo X Chen T Zhong M Jiang X Zhang L Ren C . Differential regulation of hepatopancreatic vitellogenin (Vtg) gene expression by two putative molt-inhibiting hormones (MIH1/2) in Pacific white shrimp (Litopenaeus vannamei). Peptides. (2015) 68:58–63. doi: 10.1016/j.peptides.2014.11.002 25447412

[24] Chen T Zhang L-P Wong N-K Zhong M Ren C-H Hu C-Q . Pacific white shrimp (Litopenaeus vannamei) vitellogenesis-inhibiting hormone (VIH) is predominantly expressed in the brain and negatively regulates hepatopancreatic vitellogenin (Vtg) gene expression. Biol Reprod. (2014) 90:47. doi: 10.1095/biolreprod.113.115030 24451988

[25] Chen T Wong N-K Jiang X Luo X Zhang L Yang D . Nitric oxide as an antimicrobial molecule against Vibrio harveyi infection in the hepatopancreas of Pacific white shrimp, Litopenaeus vannamei. Fish Shellfish Immunol. (2015) 42:114–20. doi: 10.1016/j.fsi.2014.10.042 25449376

[26] Liu Z Liu J Liu Z Song X Liu S Liu F . Identification and characterization of a novel insulin-like receptor (LvRTK2) involved in regulating growth and glucose metabolism of the Pacific white shrimp Litopenaeus vannamei. Biomolecules. (2024) 14:1300. doi: 10.3390/biom14101300 39456233 PMC11506343

[27] Liu J Liu Z Liu Z Song X Liu S Song L . Identification and characterization of a novel type (insulin-type) of insulin-like peptide involved in glucose metabolism and growth in Pacific white shrimp Litopenaeus vannamei. Aquaculture. (2026) 612:743162. doi: 10.1016/j.aquaculture.2025.743162 42574925

[28] Livak KJ Schmittgen TD . Analysis of relative gene expression data using real-time quantitative PCR and the 2^-ΔΔ^CT method. Methods. (2001) 25:402–8. doi: 10.1006/meth.2001.1262 11846609

[29] Chung JS . An insulin-like growth factor found in hepatopancreas implicates carbohydrate metabolism of the blue crab Callinectes sapidus. Gen Comp Endocrinol. (2014) 199:56–64. doi: 10.1016/j.ygcen.2014.01.012 24503150

[30] Luffy M Ganz A-L Wagner S Wolf J Ropertz J Zeidan R . Linsitinib inhibits proliferation and induces apoptosis of both IGF-1R and TSH-R expressing cells. Front Immunol. (2024) 15:1488220. doi: 10.3389/fimmu.2024.1488220 39723207 PMC11668815

[31] Mulvihill MJ Cooke A Rosenfeld-Franklin M Buck E Foreman K Landfair D . Discovery of OSI-906: a selective and orally efficacious dual inhibitor of the IGF-1 receptor and insulin receptor. Future Med Chem. (2009) 1:1153–71. doi: 10.4155/fmc.09.89 21425998

[32] Gao Y Zhang X Zhang X Yuan J Xiang J Li F . CRISPR/Cas9-mediated mutation reveals Pax6 is essential for development of the compound eye in Decapoda Exopalaemon carinicauda. Dev Biol. (2020) 465:157–67. doi: 10.1016/j.ydbio.2020.07.001 32702356

[33] Si S Zhang X Yu Y Zhang X Zhong X Yuan J . Structure and function analyses of the Mmd2 gene in Pacific white shrimp Litopenaeus vannamei. Front Genet. (2023) 14:1151193. doi: 10.3389/fgene.2023.1151193 37485334 PMC10361620

[34] Pang Y Zhang X Yuan J Zhang X Su M Li F . Glycogen synthase kinase 3 gene is important in growth and molting of the Pacific white shrimp Litopenaeus vannamei. Front Mar Sci. (2021) 8:681966. doi: 10.3389/fmars.2021.681966

[35] Liu F Shi W Ye H Liu A Zhu Z . RNAi reveals role of insulin-like androgenic gland hormone 2 (IAG2) in sexual differentiation and growth in hermaphrodite shrimp. Front Mar Sci. (2021) 8:666763. doi: 10.3389/fmars.2021.666763

[36] Jayesh P Jose S Philip R Bright Singh IS . A novel medium for the development of *in vitro* cell culture system from Penaeus monodon. Cytotechnology. (2013) 65:307–22. doi: 10.1007/s10616-012-9491-9 23053784 PMC3597175

[37] Florea A Ho S Mee J Houston R Robledo D Bean TP . Towards new horizons in shrimp cell culture: a streamlined protocol for culturing healthy, enduring haemocytes and lymphoid organ cells from Pacific whiteleg shrimp (Litopenaeus vannamei). Aquaculture. (2026) 613:743279. doi: 10.1016/j.aquaculture.2025.743279 42574925

[38] Sivakumar S Kalaimani N . An optimization of supplements and physical factors for growth of hemocytes culture from Penaeus vannamei (white shrimp) in selective medium. Mol Biol Rep. (2022) 49:9489–97. doi: 10.1007/s11033-022-07834-y 36006504

[39] Musgrove L Russell FD Ventura T . Considerations for cultivated crustacean meat: potential cell sources, potential differentiation and immortalization strategies, and lessons from crustacean and other animal models. Crit Rev Food Sci Nutr. (2024) 65:2431–55. doi: 10.1080/10408398.2024.2342480 38733287

[40] Kemeh MM Lazo ND . Modulation of the activity of the insulin-degrading enzyme by Aβ peptides. ACS Chem Neurosci. (2023) 14:2935–43. doi: 10.1021/acschemneuro.3c00384 37498802

[41] Leissring M González-Casimiro C Merino B Suire C Perdomo G . Targeting insulin-degrading enzyme in insulin clearance. Int J Mol Sci. (2021) 22:2235. doi: 10.3390/ijms22052235 33668109 PMC7956289

[42] Ricort JM Tanti JF Van Obberghen E Le Marchand-Brustel Y . Alterations in insulin signalling pathway induced by prolonged insulin treatment of 3T3-L1 adipocytes. Diabetologia. (1995) 38:1148–56. doi: 10.1007/BF00422363 8690166

[43] Turner MC Martin NRW Player DJ Ferguson RA Wheeler P Green CJ . Characterising hyperinsulinemia-induced insulin resistance in human skeletal muscle cells. J Mol Endocrinol. (2020) 64:125–32. doi: 10.1530/jme-19-0169 31990657

[44] Jayesh P Seena J Singh ISB . Establishment of shrimp cell lines: perception and orientation. Indian J Virol. (2012) 23:244–51. doi: 10.1007/s13337-012-0089-9 23997447 PMC3550748

[45] Toullec J-Y . Crustacean primary cell culture: a technical approach. Methods Cell Sci. (1999) 21:193–8. doi: 10.1023/A:1009833924792 10627671

[46] Rinkevich B Pomponi SA . Advancing marine invertebrate cell line research: four key knowledge gaps. In Vitro Cell Dev Biol Anim. (2025) 61:493–505. doi: 10.1007/s11626-025-01029-y 40153206 PMC12245970

[47] Zhang X Dong D Guo H Han Q Lu Q Liang C . Problems with the use of liposome- and retrovirus-mediated gene transfer methods in the primary lymphoid cells of the oka organs of the greasyback shrimp, Metapenaeus ensis (De Haan, 1844). Crustaceana. (2015) 88:1351–65. doi: 10.1163/15685403-00003498

[48] Jiang Y-S Zhan W-B Wang S-B Xing J . Development of primary shrimp hemocyte cultures of Penaeus chinensis to study white spot syndrome virus (WSSV) infection. Aquaculture. (2006) 253:114–9. doi: 10.1016/j.aquaculture.2005.07.045 42574925

[49] McKinley ET Bugaj JE Zhao P Guleryuz S Mantis C Gokhale PC . 18FDG-PET predicts pharmacodynamic response to OSI-906, a dual IGF-1R/IR inhibitor, in preclinical mouse models of lung cancer. Clin Cancer Res. (2011) 17:3332–40. doi: 10.1158/1078-0432.Ccr-10-2274 21257723 PMC3122480

[50] Jose S Mohandas A Philip R Bright Singh IS . Primary hemocyte culture of Penaeus monodon as an *in vitro* model for white spot syndrome virus titration, viral and immune related gene expression and cytotoxicity assays. J Invertebr Pathol. (2010) 105:312–21. doi: 10.1016/j.jip.2010.08.006 20807537

[51] Al-Mohanna SY Nott JA . B-cells and digestion in the hepatopancreas of *Penaeus semisulcatus* (Crustacea: Decapoda). J Mar Biol Assoc U K. (1986) 66:403–14. doi: 10.1017/S0025315400043034 41292463

[52] Jacobs W . Untersuchungen über die Cytologie der Sekretbildung in der Mitteldarmdrüse von *Astacus leptodactylus*. Z Zellforsch Mikrosk Anat. (1928) 8:1–62. doi: 10.1007/BF02658699 30311153

[53] Aldersey JE Abernathy JW Lange MD García JC Shoemaker CA Beck BH . Single-cell transcriptomics of Pacific white shrimp (*Litopenaeus vannamei*) hepatopancreas reveal immune and metabolic responses to AHPND-causing Vibrio parahaemolyticus. Front Immunol. (2026) 17:1713369. doi: 10.3389/fimmu.2026.1713369 41694335 PMC12902777

[54] Florea A Daniels RR Salisbury SJ Furniss J Robledo D Bean TP . Generation of a *Litopenaeus vannamei* hepatopancreas cell atlas from single nuclei transcriptomics using a new nuclei isolation method. BMC Genomics. (2025) 27:112. doi: 10.1186/s12864-025-12475-z 41455910 PMC12860103

[55] Li R Tian JZ Wang MR Zhu LN Sun JS . EsGLUT4 and CHHBP are involved in the regulation of glucose homeostasis in the crustacean *Eriocheir sinensis*. Biol Open. (2017) 6:1279–89. doi: 10.1242/bio.027532 28751307 PMC5612244

[56] Trapp M Valle SC Pöppl AG Chittó ALF Kucharski LC Da Silva RSM . Insulin-like receptors and carbohydrate metabolism in gills of the euryhaline crab *Neohelice granulata*: effects of osmotic stress. Gen Comp Endocrinol. (2018) 262:81–9. doi: 10.1016/j.ygcen.2018.03.017 29548758

[57] Calvo NS Stumpf L Cortés-Jacinto E Castillo Díaz F López Greco LS . Mobilization of energetic reserves during starvation in juveniles of different size of the redclaw crayfish *Cherax quadricarinatus*. Aquac Nutr. (2018) 24:952–60. doi: 10.1111/anu.12631 40046247

[58] Gulbins A Horstmann M Daser A Flögel U Oeverhaus M Bechrakis NE . Linsitinib, an IGF-1R inhibitor, attenuates disease development and progression in a model of thyroid eye disease. Front Endocrinol. (2023) 14:1211473. doi: 10.3389/fendo.2023.1211473 37435490 PMC10331459

[59] Vella V Nicolosi ML Giuliano M Morrione A Malaguarnera R Belfiore A . Insulin receptor isoform A modulates metabolic reprogramming of breast cancer cells in response to IGF2 and insulin stimulation. Cells. (2019) 8:1017. doi: 10.3390/cells8091017 31480557 PMC6770491

[60] Xu R Wang M Tu S Xie X Zhu D . Molecular identification of an insulin-like peptide from the swimming crab *Portunus trituberculatus* and evidence for its glucoregulation function. Front Mar Sci. (2023) 10:1144781. doi: 10.3389/fmars.2023.1144781

[61] Jiang Q Jiang Z Gu S Qian L Li X Gao X . Insights into carbohydrate metabolism from an insulin-like peptide in *Macrobrachium rosenbergii*. Gen Comp Endocrinol. (2020) 293:113478. doi: 10.1016/j.ygcen.2020.113478 32243957

[62] Nagai C Nagata S Nagasawa H . Effects of crustacean hyperglycemic hormone (CHH) on the transcript expression of carbohydrate metabolism-related enzyme genes in the kuruma prawn, *Marsupenaeus japonicus*. Gen Comp Endocrinol. (2011) 172:293–304. doi: 10.1016/j.ygcen.2011.03.019 21447337

[63] Ao S Xu W Ding Q Gao X Zhang X Jiang Q . Regulation of glucose homeostasis in *Macrobrachium rosenbergii*: effects of glucose load on glucose metabolism and hormonal response. Aquaculture. (2023) 577:739902. doi: 10.1016/j.aquaculture.2023.739902 42574925

[64] Wang ZQ Yu Y Zhang XH Komorowski J . Chromium–insulin reduces insulin clearance and enhances insulin signaling by suppressing hepatic insulin-degrading enzyme and proteasome protein expression in KKAY mice. Front Endocrinol. (2014) 5:99. doi: 10.3389/fendo.2014.00099 25071716 PMC4083453

[65] Kellon EM . Insulin resistance versus dysregulation—a distinction without a difference. Equine Vet Educ. (2025) 37:210–5. doi: 10.1111/eve.14082 40046247

[66] Hamza SM Sung MM Gao F Soltys CM Smith NP MacDonald PE . Chronic insulin infusion induces reversible glucose intolerance in lean rats yet ameliorates glucose intolerance in obese rats. Biochim Biophys Acta Gen Subj. (2017) 1861:313–22. doi: 10.1016/j.bbagen.2016.11.029 27871838

[67] Zakout Y-A Abdellah MA Abdallah MA Batran SA . Optimization of PAS stain and similar Schiff's based methods for glycogen demonstration in liver tissue. Histochem Cell Biol. (2024) 161:359–64. doi: 10.1007/s00418-023-02261-x 38147127

[68] Mehlem A Hagberg CE Muhl L Eriksson U Falkevall A . Imaging of neutral lipids by Oil Red O for analyzing the metabolic status in health and disease. Nat Protoc. (2013) 8:1149–54. doi: 10.1038/nprot.2013.055 23702831

